# The lung-brain axis mediates the neuroprotective effects of nasally administered *L. salivarius* and its EV-delivered metabolite in vascular dementia

**DOI:** 10.1186/s12974-026-03864-6

**Published:** 2026-05-12

**Authors:** Cihua Zheng, Zhuoya Wang, Furui Tang, Yuchun Zhong, Jiacheng Zheng, Jian Xie, Li Liu, Yimin Pi, Xifeng Wang, Tian Liu, Zhidong He, Jun Luo

**Affiliations:** 1 Department of Rehabilitation Medicine, The Second Affiliated Hospital, Jiangxi Medical College, Nanchang University, Nanchang, Jiangxi 330006 P. R. China; 2 Jiangxi Province Key Laboratory of Precision Cell Therapy, The Institute of Translational Medicine, The Second Affiliated Hospital of Nanchang University, Nanchang, Jiangxi 330006 P. R. China; 3 Department of Traditional Chinese Medicine, The Second Affiliated Hospital, Jiangxi Medical College, Nanchang University, Nanchang, Jiangxi 330006 P. R. China; 4 Department of Anesthesiology, the First Affiliated Hospital, Jiangxi medical College, Nanchang University, Nanchang, Jiangxi 330006 P. R. China; 5 Department of Orthopedics, Longyan First Hospital, Longyan, Fujian 364000 PR China

**Keywords:** Vascular dementia, *L. salivarius*, Bacterial extracellular vesicles, Asperuloside

## Abstract

Neuroinflammation and impaired barrier function are two prominent pathological mechanisms contributing to cognitive impairment in patients with vascular dementia (VaD). Currently, effective treatments for VaD remain limited, underscoring the clinical significance of developing novel, multi-targeted therapeutic strategies. In recent years, more and more studies have shown the connection between lung and brain, so we used nasal administration of probiotics to observe the improvement of cognitive function in VaD rats. Because the safety of the organism is uncertain, the study develop a bacterial extracellular vesicles (EVs) drug delivery system that delivers the key bioactive metabolite asperuloside (ASP) by modulating the microbiota–lung–brain axis, aiming to improve brain targeting and therapeutic outcomes. The results show that nasal administration of *L. salivarius* significantly ameliorated cognitive impairment, mitigated neuroinflammation, restored blood-brain barrier and lung barrier function, and modulated lung flora in VaD rats. Metabolomics analysis identified ASP as the principal active metabolite, although its efficacy as a standalone agent was constrained. The EA system effectively facilitated ASP delivery to brain tissue, yielding neuroprotective and barrier-repair effects. Collectively, our study shows that *L. salivarius* can modulate the pathophysiological processes of VaD via the “microbiota-lung-brain axis.” Its EVs serve as effective vehicles for delivering active metabolites, offering a novel integrated therapeutic approach for VaD involving microbial metabolism delivery.

**Supplementary Information:**

The online version contains supplementary material available at 10.1186/s12974-026-03864-6.

## Introduction

Vascular dementia (VaD) is the second most common type of dementia after Alzheimer’s disease. It is caused by abnormal cerebral vascular structure or function, and its main clinical manifestation is progressive decline of cognitive function [1]. As the global population ages, VaD incidence continues to rise and has become a major public health problem. The pathological mechanism of this disease is complex, involving multiple interrelated biological processes such as chronic cerebral hypoperfusion, blood-brain barrier destruction, neuroinflammatory activation, oxidative stress and neuronal apoptosis [2]. Currently, clinical treatment methods for VaD lack efficacy. Available medications offer only temporary symptom relief, without the ability to halt disease progression, often leading to notable side effects [3]. Hence, investigating VaD pathogenesis and innovating therapeutic approaches hold paramount importance.

There is increasing evidence that cognitive impairment occurs more frequently in patients with chronic lung disease than in those without lung disease. Chronic obstructive pulmonary disease (COPD) affects 210 million people, 60% of whom have cognitive impairment [4]. Patients with chronic lung disease are thought to be at higher risk for cognitive decline. Since harmful factors can affect cognitive function through the lungs, can we protect cognitive function by administering beneficial factors? In recent years, the concept of a “microbiota-lung-brain axis” has garnered increasing attention [5]. Research indicates that compromised lung function and dysbiosis in the pulmonary microbiota can impact the central nervous system by modulating immune responses and inflammatory pathways, thereby influencing the progression of neurodegenerative conditions [6]. Previous study has shown that cigarette smoke destroys lung barrier function and increases BBB permeability, causing inflammatory factors to enter the brain through the lung brain axis, triggering neuroinflammation, damaging hippocampal neuronal and synaptic function, and eventually triggering neurocognitive dysfunction [7]. In addition, neomycin administration alters the pulmonary microbiota and alleviates migraine via the BDNF/TrkB pathway [8]. Therefore, it is feasible to improve neurocognitive function by affecting the lung microbiome. Intranasal drug delivery, as a non-invasive drug delivery method, has the advantages of avoiding first-pass effect, strong brain targeting and good patient compliance, and has gradually become a research hotspot of drug delivery in central nervous system [9]. *Lactobacillus*, a significant probiotic genus, plays a pivotal role in immune regulation, inflammation mitigation, and metabolic enhancement [10]. Recent studies have found that nasal administration of certain *Lactobacillus* strains (such as *L. rhamnosus* and *L. johnsonii*) can regulate lung microbial composition, alleviate airway inflammation, and indirectly affect distal organ function, providing theoretical basis for the application of nasal probiotic intervention in neurological diseases [11, 12]. However, *L. salivarius* has demonstrated the ability to protect dopaminergic neurons, alleviate oxidative stress, and reduce neuroinflammation, thereby exerting neuroprotective effects in models of Parkinson’s Disease [13]. Nevertheless, the utilization of this strain in VaD, particularly through nasal administration, remains inadequately explored. Based on this, we propose the scientific hypothesis that nasal administration of *L. salivarius* may improve neuroinflammation and cognitive dysfunction in VaD by regulating the microbiota-lung-brain axis.

Despite the potential of viable bacteria therapy, its application still faces multiple challenges such as stability, safety and dose control [14]. Bacterial extracellular vesicles (EVs), as natural nano-carriers, not only have good biocompatibility and low immunogenicity, but also can carry bioactive components of parent bacteria, penetrate blood-brain barrier and target pathological regions [15]. In recent years, EVs have become the research frontier of drug delivery system. Several studies have shown that EVs derived from *Lactobacillus* can play a therapeutic role in models such as neuroinflammation and autism [16, 17]. Thus, EV-based drug delivery strategies represent a promising direction for overcoming the current limitations of live bacterial therapies.

In our study, it was found that the ASP content in brain tissue was significantly higher after *L. salivarius* treatment than in VaD rats. In addition, ASP is an iridoid with multiple pharmacological effects including anti-inflammatory, anticancer, neuroprotective and anti-obesity [18]. Studies have shown that ASP exhibits beneficial effects in a variety of disease models, such as alleviating lipopolysaccharide (LPS)-induced acute lung injury, improving depression-like behavior in rats, reducing food intake, body weight and fat mass in rats fed a high fat diet, and inhibiting tumor growth [18–21]. However, its direct application still has some limitations, such as poor stability, low brain distribution efficiency and high dose to maintain efficacy [22].

Based on the aforementioned background, we devised a three-phase study. Initially, we aimed to assess the impact of intranasal administration of *L. salivarius* on cognitive function, neuroinflammation, and lung microbiota in rats with VaD. Subsequently, we sought to identify and confirm the therapeutic efficacy of the principal bioactive compound, ASP, through metabolomic analysis. Lastly, we aimed to address the challenge of efficient delivery by developing a nanoformulation of EVs encapsulating ASP sourced from *L. salivarius*. This formulation aims to enable precise, high-efficiency, low-dose nasal therapy targeting VaD. The primary objective of this investigation is to propose an innovative therapeutic approach for VaD leveraging a microbial metabolism-based delivery system and to furnish fresh empirical support for understanding the regulatory mechanisms of the microbiota-lung-brain axis.

## Materials and methods

### Animal experiment

SPF Sprague-Dawley (SD) rats, aged 6–8 weeks and weighing 180–220 g, were procured from Tianqin Biotechnology Co., Ltd., located in Changsha City, Hunan Province. The rats were acclimated for one week at 22 °C under a 12-hour light-dark cycle, with ad libitum access to food and water.

VaD rat model induced by chronic cerebral ischemia was established by ligating bilateral common carotid artery (2-VO). The procedure involved the anesthesia of rats with ether, followed by skin preparation and disinfection in the anterior cervical region. A 5 mm skin incision was made on the right side of the cervical median line to expose the right common carotid artery. Care was taken to avoid traction on the vagus nerve during blunt separation and exposure of the artery. The skin was sutured after ligation, and warm resuscitation was performed. After 48 h, the left common carotid artery was ligated using the same method [23]. Twenty-four rats were randomly allocated into three groups (*n* = 8/group): the control group (CON) received normal feeding; the model group (MOD) underwent nasal instillation of 100 µL physiological saline every other day. Each animal received 100 µL of formulation per nostril (total 200 µL per animal), administered via slow, alternating instillation, once every other day, for a total of 15 doses over a 30-day treatment period. the *L. salivarius* treatment group (LST) received nasal instillation of 100 µL of 1 × 10^8^ CFU/mL *L. salivarius* suspension every other day [24]. To investigate the impact of ASP on VaD, an ASP treatment group was included: ASP (2 mg/kg) suspension was administered nasally every other day after model establishment. Thirty-two rats were randomly divided into four groups (*n* = 8/group): the CON, MOD, LST and ASP group. To enhance ASP delivery efficiency, EVs derived from *L. salivarius* were utilized to encapsulate ASP, namely EVs-ASP, and the intervention effect was assessed. Forty rats were randomly divided into five groups (*n* = 8/group): the CON group received normal feeding; the MOD group underwent nasal instillation of 100 µL saline every other day; the LST group received *L. salivarius*; the EVs group (EV) received nasal instillation of EVs (100 µg) every other day; the EVs-ASP group (EA) received nasal instillation of EVs loaded with ASP (100 µg EVs, ASP approximately 2 mg/kg; 25% drug loading rate, ASP concentration approximately 0.4762 mg). every other day. Following 30 days of continuous intervention, cognitive function was evaluated through behavioral tests, and subsequent sample collection was conducted for further analysis [25].

### Extraction of bacterial EVs

Cultivate and count *L. salivarius*, take the bacterial solution and centrifuge twice (30 min each time) at 4 °C and 5000 g to obtain the supernatant. The supernatant is filtered through 0.45 μm and 0.22 μm filters respectively, and then concentrated using a 100 kDa Amicon Ultra-15 filter at 5000×g for 30 min at 4℃ [26]. The concentrated solution is centrifuged at 4 °C and 150,000×g for 90 min, and the supernatant is discarded to obtain EVs containing a small amount of impurities; When purifying, wash the precipitate three times with sterile PBS and resuspend it well [27].

### EA drug loading

1 mg ASP and diluted EV were mixed ultrasonically in ice bath, and the parameters were set to 500 V, 2 kHz and 20% power. Six cycles were treated in pulse mode of 2 s on /2 s off, and cooled on ice for 2 min between cycles. After ultrasound, the samples were incubated at 37 °C for 2–3 h to recover the structure, and then centrifuged at 12,000 g for 10 min to remove the unloaded drugs. The content of ASP in EA was determined by ultraviolet spectrophotometry at 238 nm, and the standard curve was established with 0.0078–0.125 mg/mL ASP standard solution for calculation. The encapsulation efficiency is calculated according to the formula EE = We/Wt × 100%, and the drug loading is calculated according to LE = We/(Wt + Ws) × 100%, where Wt is the total amount of ASP, We is the quality of ASP loaded, and Ws is the quality of EV protein estimated by BCA method.

### Behavioral experiments

Cognitive function and emotional behaviors in animal models of nervous system diseases were evaluated using the three-chamber social test, open field test, Morris water maze, elevated plus maze, and sucrose preference test.

### Quantitative Polymerase Chain Reaction (qPCR)

Take rat brain tissue homogenate, extract total RNA with TRIzol reagent (Invitrogen), then reverse transcribe RNA into cDNA with all-in-one first strand cDNA synthesis kit II (with dsDNA SE). Next, according to the instructions of PCR kit, amplification was carried out in PCR instrument by three-step method [28].

The primer sequences required for rat target genes and internal reference genes are shown below.

**Table:** 

Name	primer sequence
TNF-α-Forward Primer	CCACCACGCTCTTCTGTCTACTG
TNF-α-Reverse Primer	TGGGCTACGGGCTTGTCACT
IL-6-Forward Primer	AGGATACCACCCACAACAGACC
IL-6-Reverse Primer	TTGCCATTGCACAACTCTTTTC
IL-1β-Forward Primer	TGACCTGTTCTTTGAGGCTGAC
IL-1β-Reverse Primer	CATCATCCCACGAGTCACAGAG
GAPDH-Forward Primer	CTGGAGAAACCTGCCAAGTATG
GAPDH-Reverse Primer	GGTGGAAGAATGGGAGTTGCT

### Western blot

Western blot was conducted as per the steps outlined in this document [29].

### Golgi staining

Rat brain tissue was fixed in Golgi staining fixative (Servicebio G1069) and stored at room temperature. The fixative was then replaced with Gorky dye solution, and the tissue was stained at 26 °C for 14 days. The dye solution was changed 48 h after initiation of staining and subsequently every 3 days. Following staining, the brain tissue underwent a 1-hour treatment in tissue treatment solution, followed by a 3-day treatment at 4 °C in darkness with fresh solution. The tissue was then sectioned into 60 μm slices using a vibrating microtome and mounted on glass slides. The slices were rinsed with ultrapure water, treated with developer for 30 min, rinsed again, sealed with glycerol gelatin, and stored in darkness. Golgi morphology was observed using an optical microscope, and images were captured [30].

### ELISA

Peripheral serum of rats was collected and centrifuged at 4 °C and 1000×g for 15 min. Then, according to the kit instructions, the concentrations of IL-1β, TNF-α and IL-6 in serum were quantitatively detected by ELISA kits (IL-1β: Cat# RK00009; IL-6: Cat# RK00020; TNF-α: Cat#RK00029; all purchased from ABClonal, China).

### DNA extraction and 16 S rRNA gene sequencing

The frozen lung tissue was taken out and the total gene DNA of rat lung microorganism was extracted according to the instructions of genome DNA kit. The variable V3-V4 region of the 16 S rRNA gene (338 F, 5′- ACTCCTACGGGAGGCAGCA-3′; 806R, 5′- GGACTACHVGGGTWTCTAAT-3 ′) was amplified for each sample using primers 338 F/806R. The PCR products were sequenced using Illumina platform and aligned with known 16 S rRNA gene databases to determine the microbial species to which the sequences belonged. According to the results, the relative abundance of different microorganisms in the samples was counted to analyze the composition structure of microbial community in rat lung tissue, and the α diversity index and β-diversity index were calculated to evaluate the diversity of microbial community in rat lung tissue and the difference between samples [31].

### Untargeted metabolomics

Weigh 50 milligrams of rat brain and lung tissue samples to prepare the required sample solution. The sample was then sent to Parthenol Biotech (Shanghai) for GC-TOF-MS analysis using a DB-5MS column with a flow rate of 1 mL/min. A 1 µL sample was injected at a 1:10 split ratio with an injection port temperature of 280 °C. The transmission line and ion source temperatures were set at 320 °C and 230 °C, respectively. The temperature program started at 50 °C for 30s, followed by a ramp of 15 °C/min to 320 °C, which was maintained for 9 min. Mass spectrometry was performed at a rate of 10 spectra/s with an electron energy of -70 V and a 3-minute solvent delay for full scan. Subsequent experiments and bioinformatics analyses were conducted upon sample qualification [27].

### Targeted metabolomic analysis of SCFAs

Rat brain and lung tissues were rapidly frozen in dry ice. A gradient mixture containing seven short-chain fatty acid standards (SCFAs) was prepared to establish a standard curve based on the chromatogram. Approximately 50 mg of the quick-frozen tissue was homogenized after grinding, followed by ultrasonication in an ice-water bath for 30 min. The homogenate was then centrifuged at 4℃ and 10,000×g for 15 min to collect the supernatant. Subsequently, ethyl acetate was added to the supernatant, vortex mixed, ultrasonicated in an ice-water bath for 10 min, and centrifuged at 4℃ and 10,000×g for 10 min. The resulting supernatant was analyzed for the concentrations of the seven SCFAs using gas chromatography-mass spectrometry [32] .

### RNA-seq analysis

Total RNA was extracted from rat brain tissues in the MOD and LST groups. Library preparation and sequencing were conducted by Personalbio Biotechnology Co., Ltd. High-throughput sequencing data were obtained and aligned against reference databases to identify transcripts. Transcript data were functionally annotated using the Gene Ontology (GO), Kyoto Encyclopedia of Genes and Genomes (KEGG), Enzyme Commission (EC), EggNOG, and UniProt databases. After data normalization, differential gene expression analysis was carried out. Transcripts with a *P*-value < 0.05 and fold change (FC) > 1.5 were considered statistically significant and used as the primary criteria for identifying differentially expressed genes [33].

### Statistical analysis

The data analysis and processing of this study are all completed by GraphPad Prism 9.0 software. The data are expressed as mean ± SD. For the comparison between groups, One-Way ANOVA was used. The significance level is set to *P* < 0.05, which means that there is a statistical difference. The following symbols are used in the result chart to indicate the significance level of the difference: *P < 0.05, **P < 0.01, ***P < 0.001 [27].

Other materials and methods, including in vitro assays and in vivo studies, are provided in the Supporting Information.

## Results

### Intranasal instillation of *L. salivarius* improves cognitive dysfunction in VaD rats

**Fig. 1:**
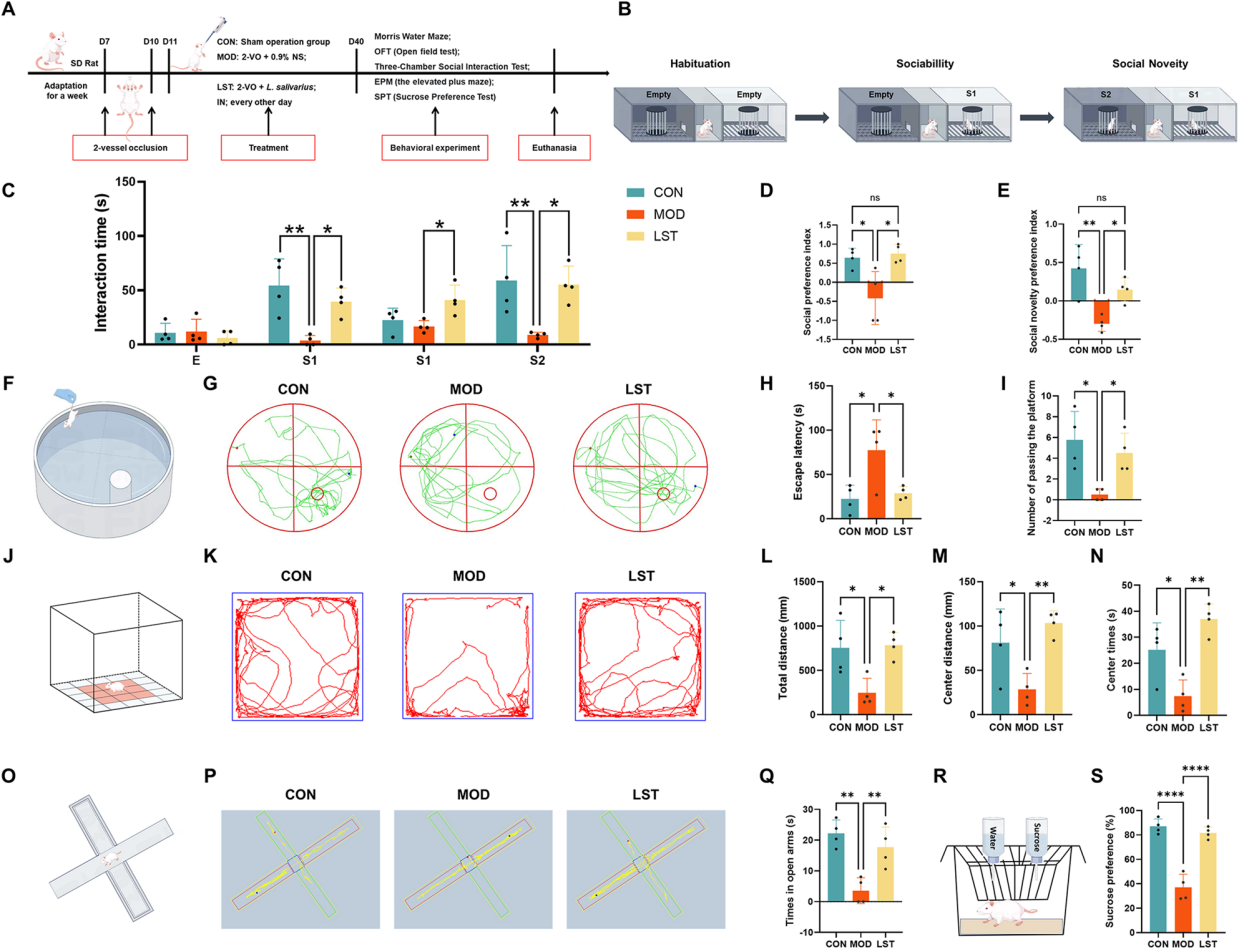
*L. salivarius* ameliorates cognitive deficits and anxiety-like behaviors in rats with VaD. **A** Schematic diagram of the experimental timeline. **B** Schematic of the three-chamber social test apparatus. **C** Time spent in the three chambers. **D** The social preference index (SPI) and (**E**) social novelty preference index (SNPI). **F** Schematic of the Morris water maze. **G** Representative movement trajectories of rats from each group during the probe trial. **H** Escape latency during training. **I** Number of crossings over the former platform location. **J** Schematic of the open field test. **K** Representative movement trajectories from the open field test. **L** Total distance traveled. **M** Distance traveled in the center area. **N** Time spent in the center area. **O** Schematic of the elevated plus maze. **P** Representative movement trajectories. **Q** Time spent in the open arms. **R** Schematic of the sucrose preference test. **S** Sucrose preference ratio. CON, normal control. MOD: VaD rats treated with normal saline every other day. LST: VaD rats administered *L. salivarius* every other day. Data are presented as mean ± SEM, *n* = 4 per group. *P < 0.05, **P < 0.01, ***P < 0.001

To explore the role of *L. salivarius* on VaD rats cognitive impairment, this experiment using bilateral common carotid artery ligation (2-VO) to establish a rat cerebral hypoperfusion VaD model. The experimental procedure was illustrated in Fig. 1A. Rats were given either saline (MOD group) or a suspension of *L. salivarius* (LST group). After 30 days of continuous treatment, the adverse effects of 2-VO on the cognitive function of rats were validated using a series of behavioral tests, in conjunction with the *L. salivarius* intervention. The outcomes of a Three-Chamber social experiment revealed a significant decrease in the social preference index and social novelty preference index of the MOD group compared to the normal control group (CON group). Following intervention with *L. salivarius*, the social preference indexes of rats in the LST group notably increased. Specifically, there was a relative increase in the time spent in the social area with unfamiliar objects and a decrease in the time spent in the empty area (Fig. 1B-E). These findings suggest that *L. salivarius* can enhance social willingness, improve social behavior, and enhance social memory function in VaD rats.

To assess spatial learning and memory abilities, rats underwent the Morris water maze experiment (Fig. 1F), which included five days of hidden platform training followed by two minutes of free exploration after platform removal. During the exploration phase, rats in the CON and LST groups predominantly swam in the area where the original platform was positioned, whereas rats in the MOD group spent significantly less time in this area (Fig. 1G). Statistical analysis further supported these observations (Fig. 1H, I), indicating that after 2-VO induction, rats in the MOD group experienced prolonged escape latency (time taken to find the hidden platform) and a reduced number of crossings over the original platform. Conversely, intervention with *L. salivarius* effectively reversed these abnormal indices and mitigated spatial learning and memory deficits in VaD rats. The results of the open field test (Fig. 1J–N) showed that compared with the control group, the total walking distance and the distance from the central area of the MOD group were significantly reduced, and the time spent in the center was also significantly reduced. These results showed that spontaneous motor activity was reduced and anxiety-like behavior was obvious. On the contrary, compared with the MOD group, the rats in the LST group showed significantly increased total distance and central area distance, as well as markedly increased stay time in the central area. These findings indicated that the administration of *L. salivarius* can reduce the anxiety-like behavior, enhance the spontaneous activity and improve the exploratory behavior of VaD rats.

Plus maze was used to evaluate anxiety-like behavior and risk-safety discrimination in rats (Fig. 1O). The results (Fig. 1P, Q) showed that compared with CON group, rats in MOD group spent significantly less time on the open arms and were almost completely confined to the closed arms. In contrast, the open-arm retention time of LST group was significantly longer than that of MOD group, which was equivalent to that of CON group. These findings showed that *L. salivarius* can effectively alleviate the anxiety-like behavior of VaD rats and restore their ability to distinguish between dangerous and safe environments. Finally, sucrose preference test (Fig. 1R) was conducted to evaluate the lack of pleasure and depression-like behavior. Figure 1S illustrated that the sucrose preference ratio of the MOD group was notably lower compared to both the CON and LST groups. This suggested that chronic cerebral hypoperfusion diminishes rats’ interest in reward stimulation. Nonetheless, supplementation with *L. salivarius* notably elevated the sucrose preference rate in rats with VaD, ameliorating symptoms of anhedonia and depression.

### *L. salivarius* can also alleviate systemic inflammation in rats with VaD and alleviate neuronal damage

**Fig. 2:**
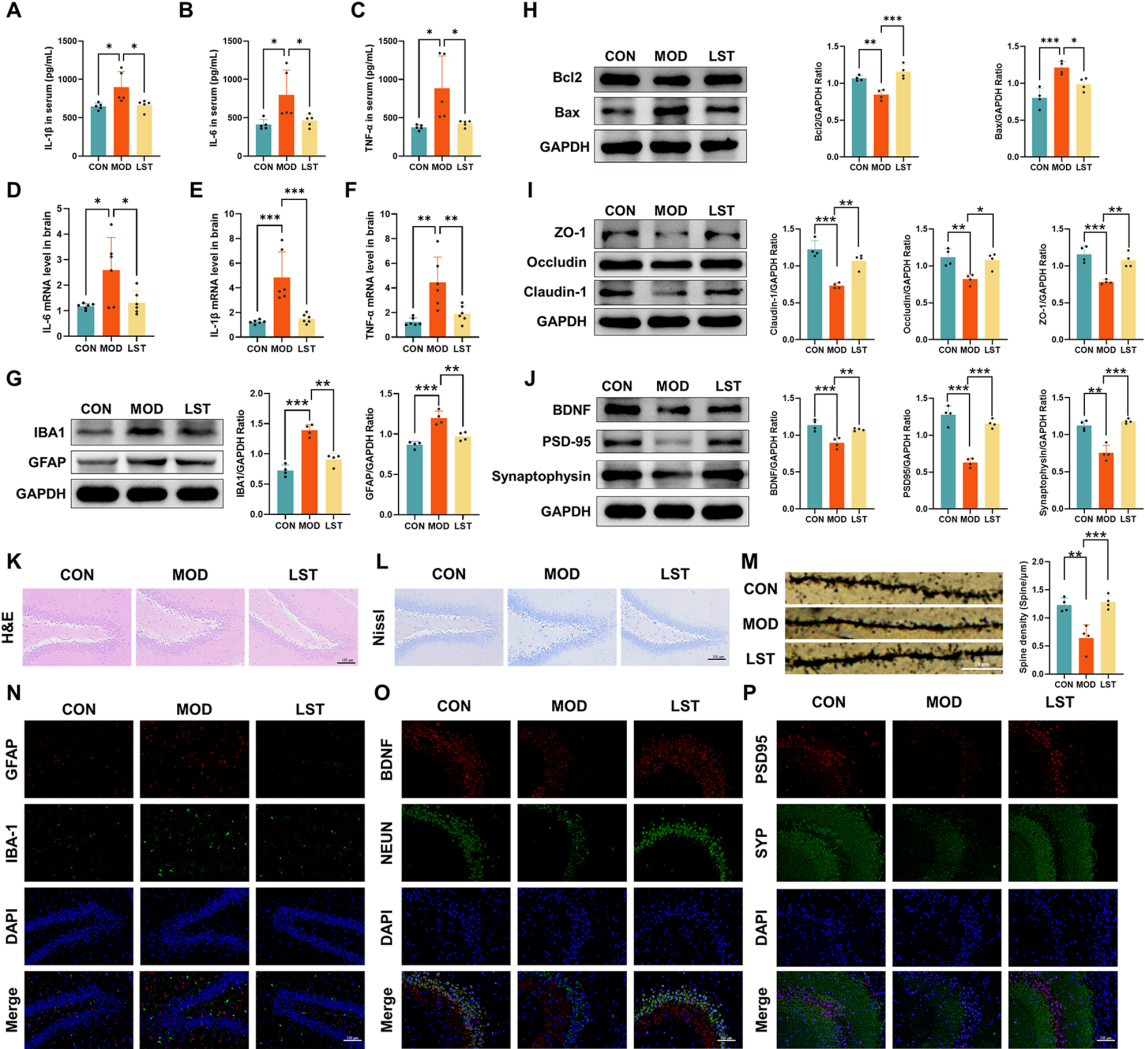
*L. salivariu*s attenuates neuroinflammation, apoptosis, and BBB disruption, and promotes neurotrophic and synaptic repair in VaD rats. **A**-**C** Serum levels of IL-6 (**A**), IL-1β (**B**), and TNF-α (**C**). **D**-**F** Brain mRNA levels of IL-6 (**D**), IL-1β (**E**), and TNF-α (**F**). **G** Protein levels of IBA-1 and GFAP. **H** Expression of apoptosis-related proteins Bax and Bcl-2. **I** Expression of tight junction proteins ZO-1, Occludin, and Claudin-1. **J** Expression of BDNF, PSD95, and Synaptophysin. **K** H&E staining of hippocampal neurons in DG area. Scale bars: 100 μm. **L** Nissl staining in DG area. Scale bars: 100 μm. **M** Golgi staining and density of dendritic spines. Scale bars: 10 μm. **N**-**P** Immunofluorescence for (**N**) IBA-1and GFAP in DG area, (**O**) NeuN and BDNF in CA3 area, (**P**) PSD95 and SYP in CA3 area. Scale bars: 100 μm. CON, normal control. MOD: VaD rats treated with normal saline every other day. LST: VaD rats administered *L. salivarius* every other day. Data are presented as mean ± SEM, *n* = 4 per group. *P < 0.05, **P < 0.01, ***P < 0.001

To further explore the effect of nasal administration of *L. salivarius* on inflammatory response and blood-brain barrier function in VaD rats, we carried out a series of experiments. The results of ELISA showed that compared with the CON group, the concentrations of inflammatory cytokines IL-6, IL-1β and TNF-α in serum of rats in MOD group were significantly increased, but the levels of these factors were significantly decreased after *L. salivarius* treatment (Fig. 2A-C). The mRNA expression levels of IL-6, IL-1β, and TNF-α in the brain tissue of the MOD group were markedly elevated compared to those of the CON group. Conversely, in the LST group, these expressions were significantly suppressed (Fig. 2D-F). Further analysis revealed that the protein expression levels of IBA-1 and GFAP-markers for microglia and astrocytes, respectively-were significantly up-regulated in the MOD group (Fig. 2G). Notably, treatment with *L. salivarius* effectively suppressed the overactivation of these two types of glial cells, thereby mitigating neuroinflammation.

To assess the impact of *L. salivarius* on apoptosis in VaD rat brain tissue, we analyzed the expression of apoptosis-related proteins. Our findings revealed a significant increase in Bax expression and a notable decrease in Bcl-2 expression in the MOD group, which was effectively reversed in the LST group (Fig. 2H). Additionally, a substantial decrease in the expression of ZO-1, Occludin, and Claudin-1 was observed in the MOD group compared to the CON group, whereas a significant increase was noted in the LST group (Fig. 2I). These results indicate that intranasal administration of *L. salivarius* effectively mitigated inflammation, suppressed apoptosis, and preserved the integrity of the blood-brain barrier in VaD rats.

We further studied the effects of *L. salivarius* on neurotrophic factors and synaptic markers in brain tissue of VaD rats. Protein expression analysis demonstrated that, in comparison to the CON group, the protein levels of BDNF, PSD95 and synaptophysin were significantly reduced in the MOD group. Notably, these reductions were markedly reversed in the LST group following treatment with *L. salivarius* (Fig. 2J). Histomorphological examination in hippocampal DG region showed that the structure of neurons in CON group was normal and arranged orderly. On the contrary, MOD group showed disordered cell arrangement, loose tissue structure and atrophy of nucleoli. The LST group showed improved cell tissue and significantly reduced morphological damage (Fig. 2K). Nissl staining in DG region further proved that Nissl bodies in MOD group were less, with vacuolar morphology, irregular distribution and pale staining. In comparison, the LST team showed a more uniform distribution of Nissl, a clear outline and structural integrity (Fig. 2L). Golgi staining showed that the synaptic density, the number of dendritic spines and the proportion of mature spines in hippocampus of VaD rats decreased. After the intervention of *L. salivarius*, these parameters were significantly improved, synaptic density increased, dendritic spines became more mature, and synaptic morphology returned to normal (Fig. 2M). Immunofluorescence staining of the hippocampal DG and CA3 regions further confirmed that the treatment of *L. salivarius* reduced neuroinflammation and contributed to the repair of ischemic nerve injury (Fig. 2N-P). Collectively, these findings prove that *L. salivarius* can effectively improve the neural structure and function of VaD rats by up-regulating the expression of neurotrophin and synapse-related proteins.

Furthermore, we assessed the lung tissue of rats in all experimental groups. HE staining revealed that the alveolar structure in all groups remained predominantly intact, with no discernible pathological alterations observed (Fig. S1A). Analysis of tight junction protein expression revealed significantly lower levels of ZO-1, Occludin, and Claudin-1 in the lung tissues of the MOD group compared to the CON group. Conversely, the expression of these proteins in the LST group was significantly upregulated in comparison to the MOD group (Fig. S1B–E). Immunofluorescence staining confirmed that in the MOD group, barrier proteins exhibited fragmented and disorganized distribution, while in the LST group, they displayed a continuous linear pattern (Fig. S1F–H). These findings collectively suggested that the intranasal administration of *L. salivarius* successfully reinstated lung barrier function in VaD rats.

### *L. salivarius* alters the microbial composition of the lungs in VaD rats

**Fig. 3:**
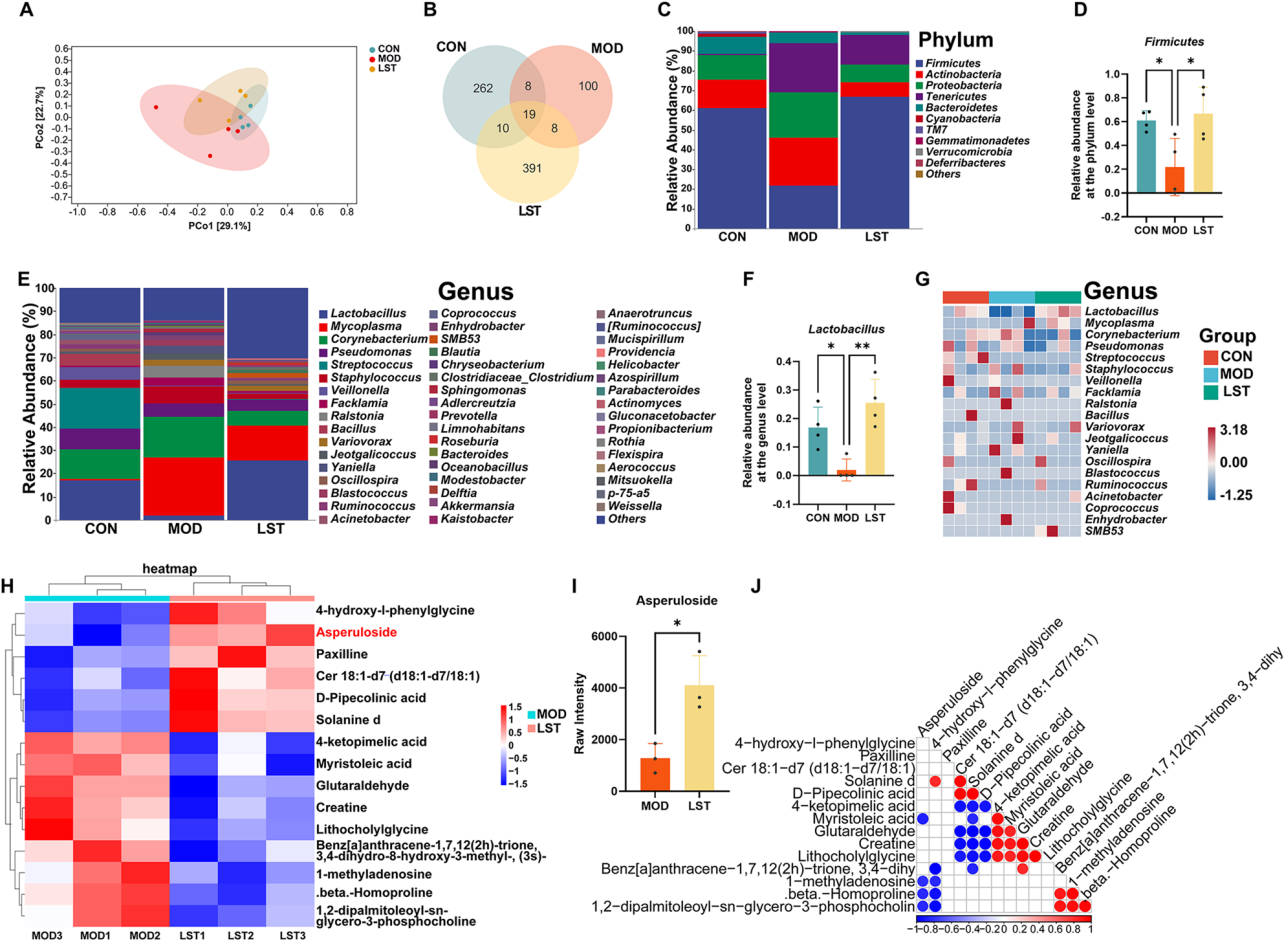
Intranasal administration of *L. salivarius* alters pulmonary microbiota and metabolite in VaD rats. **A** PCoA plot based on bacterial community structure. **B** Venn diagram of ASVs across groups. **C** Relative abundance of bacterial phyla in lung microbiota. **D** Relative abundance of *Firmicutes* at phylum level. **E** Relative abundance of bacterial genus in lung microbiota. **F** Relative abundance of *Lactobacillus* at genus level. **G** Heatmap of the relative abundance of lung microbiota at the genus level. **H** Heatmap of the pulmonary metabolites in rats from the MOD group and LST group. **I** Raw intensity of ASP. **J** Heatmap of differential metabolite associations. CON, normal control. MOD: VaD rats treated with normal saline every other day. LST: VaD rats administered *L. salivarius* every other day. Data are presented as mean ± SEM, *n* = 4 per group. *P < 0.05, **P < 0.01

The above experimental results showed that nasal administration of *L. salivarius* can improve the nervous system injury and repair the lung barrier function of VaD rats. Based on this, we further detected the microbial composition in the lung of rats, and the results showed that this administration method could change the diversity and relative abundance of pulmonary microorganisms in rats. Specifically, the results of α-diversity analysis showed that there were some differences in the pulmonary microbial diversity of rats in each group, but the differences were not statistically significant (Fig. S2A, B). The results of principal coordinate analysis (PCoA) showed that the pulmonary flora of rats in MOD group was significantly different from that in CON group in PCoA diagram, and the sample dispersion was high. However, the samples from LST group gathered in CON group, and the dispersion decreased significantly (Fig. 3A). The distribution of amplicon sequence variant (ASV) characteristic sequences in each sample was statistically analyzed, and the Venn diagram was visualized (Fig. 3B). The results showed that 299, 135 and 428 ASVs were detected in CON group, MOD group and LST group, respectively, suggesting that *L. salivarius* treatment could observably increase the total number of ASVs in rats’ lungs.

Subsequently, we analyzed the pulmonary microbial composition of each group at the classification level. At the phylum level (Fig. 3C), *Firmicutes*, *Actinobacteria* and *Proteobacteria* are the dominant phylum in the lung microbial community. Despite a significant reduction in the relative abundance of *Firmicutes* in the MOD group compared to the CON group, treatment with *L. salivarius* significantly restored its levels (Fig. 3C and D). At the genus level, compared with the rats in the CON group, the relative abundance of *Lactobacillus* in the lungs in the MOD group decreased markedly. Following intervention with *L. salivarius*, there was a notable increase in the relative abundance of this genus (Fig. 3E-G). These findings provided evidence that nasal administration of *L. salivarius* can markedly alter the pulmonary microbial composition in VaD rats.

### Effect of *L. salivarius*on the composition of metabolites in lung and brain tissues of VaD rats

To investigate the effect of intranasal administration of *L. salivarius* on the metabolic spectrum of VaD rat model, we then analyzed the metabolism of short-chain fatty acids (SCFAs) in lung and brain tissues. SCFAs detection showed that although *L. salivarius* induced some changes in the concentration of SCFA in the two tissues (Fig. S3A-I and S4A-I), there was no statistically significant difference between the two groups. Further untargeted metabolomics analysis showed that lung metabolites were mainly composed of lipids and lipid-like molecules (32.2%), organic acids and derivatives (29.5%), organic heterocyclic compounds (11.7%) and benzene ring compounds (8.7%), which together formed a metabolic spectrum (Fig. S5A). Principal component analysis (PCA) proved the obvious separation between LST group and MOD group in lung metabonomics (Fig. S5B). A total of 113 different metabolites were identified (80 up-regulated and 33 down-regulated; VIP > 1, *P* < 0.05, Fig. S5C, D). Among them, arginine-glutamine (Arg-Gln), a core metabolite, was negatively correlated with β -hyperproline (*r* =-0.94, *P* < 0.01) and positively correlated with Ser-Tyr (*r* = 0.97, *P* < 0.05), which indicated that *L. salivarius* significantly regulated VaD rats (Fig. S5E). The expanded analysis of brain tissue revealed that the metabolites similar to lung were mainly composed of lipids and lipid-like molecules (32.9%) and organic acids and derivatives (31.4%), supplemented by organic heterocyclic compounds (10.5%) and benzene ring compounds (7.4%) (Fig. S6A). PCA results showed that there are obvious differences between LST and MOD groups in brain metabonomics (Fig. S6B). The analysis of thermogram (Fig. 3H) and volcanic diagram (Fig. S6C) identified 15 different metabolites, including 9 down-regulated metabolites (such as glutaraldehyde, 1- methyladenosine; Fig. S6D–L) and 6 are up-regulated (e.g. 4- hydroxy -L- phenylglycine, ASP; Fig. 3I and S6M–Q). ASP, a core metabolite, was negatively correlated with Myristic acid (*r* =-0.88, *P* < 0.05) and positively correlated with solanine D and D-piperidinic acid (*r* = 0.99, *P* < 0.0001), which indicated that *L. salivarius* significantly regulated the metabolic characteristics in the brain of VaD rats (Fig. 3J). These findings indicated that intranasal delivery of *L. salivarius* significantly changes the metabolic composition in the lung and brain tissues of VaD rats. This cross-organ metabolic regulation may have an important impact on the outcome of the disease.

### Effect of low-dose ASP on cognitive and behavioral functions of VaD rats

**Fig. 4:**
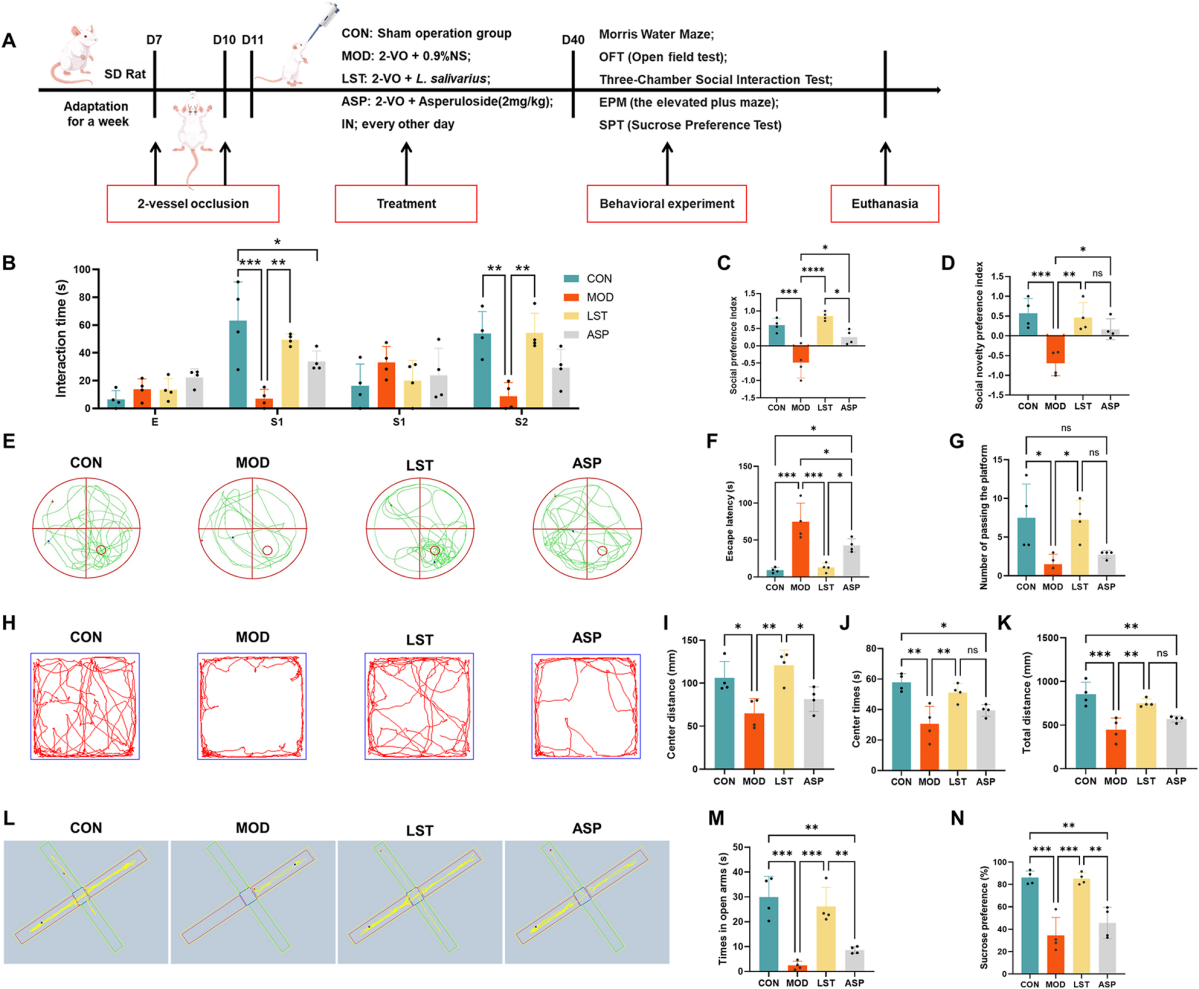
Effects of intranasal ASP on behavioral performance in VaD rats. **A** Experimental timeline. **B** Time spent in the three chambers. **C** Social preference index. **E** Social novelty preference index. **E** Representative trajectory map of the Morris water maze test. **F** Escape latency. **G** The number of platform crossings. **H** Representative movement paths. **I** Distance in center area. **J** Time in center area. **K** Total distance. **L** Representative trajectory map of the elevated plus maze test. **M** Total time spent in the open arms. **N** Sucrose preference ratio. CON, normal control. MOD: VaD rats treated with normal saline every other day. LST: VaD rats administered *L. salivarius* every other day. ASP: VaD rats administered ASP every other day. Data are presented as mean ± SEM, *n* = 4 per group. *P < 0.05, **P < 0.01, ***P < 0.001

To examine the potential cognitive benefits of ASP, a distinct metabolite found in brain tissue, on cognitive impairment in rats with VaD, post-operative VaD model rats were intranasally treated with interventions and categorized into three groups: the MOD group (receiving normal saline), the LST group (receiving *L. salivarius* suspension), and the ASP group (receiving low-dose ASP), over a 30-day intervention period (Fig. 4A). The impact of these interventions was assessed through a battery of behavioral tests. The results of three boxes of social experiments showed that the social preference index and social novelty preference index of ASP group were markedly higher than those of MOD group, but the social preference index was still lower than that of LST group (Fig. 4B-D). Morris water maze experiment showed (Fig. 4E-G) that the swimming trajectory of ASP group in the original platform area was increased compared with that of MOD group, and the escape latency during the exploration period was observably shortened, but there was no significant difference in the number of crossing the original platform. The open-field experiment showed (Fig. 4H-K) that the activity distance in the central area of ASP group was significantly lower than that of LST group, but there was no statistical difference in the activity time and total activity distance in the central area. The results of elevated plus maze (Fig. 4L, M) and sucrose preference experiment (Fig. 4N) revealed that the open arm residence time of ASP group was significantly lower than that of CON group and LST group, and the sucrose preference ratio was also lower than that of the above two groups. In conclusion, nasal administration of low-dose ASP was capable of improving behavioral abnormalities in VaD rats to some extent, but it was less effective than *L. salivarius* intervention, with its overall effect being limited.

### Limited neuroprotection but significant barrier repair by nasal ASP in VaD rats

**Fig. 5:**
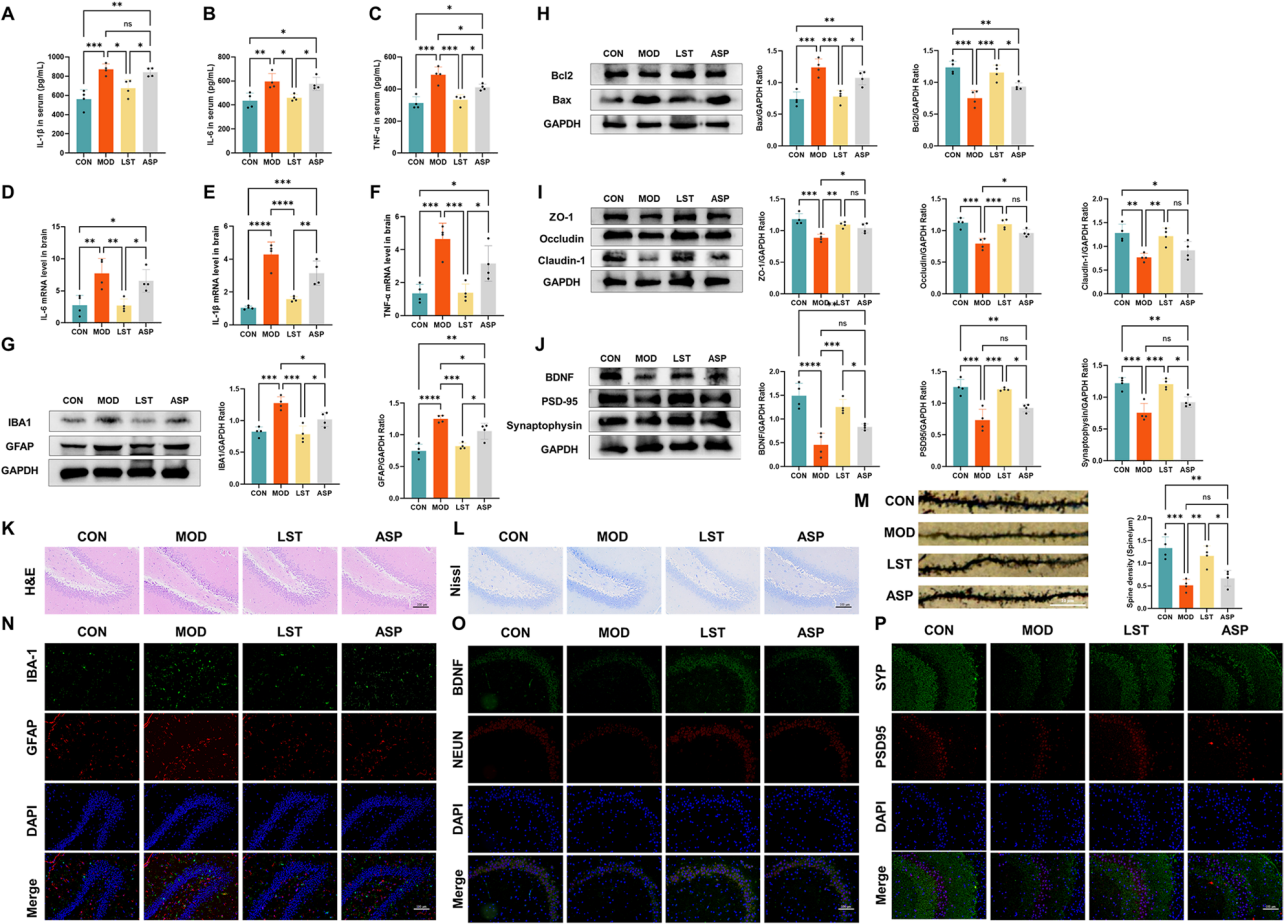
Effects of intranasal ASP on neuroinflammation and neuroprotection in VaD rats. **A**-**C** Serum levels of (A) IL-1β, (**B**) IL-6 and (**C**) TNF-α. **D**-**F** Brain mRNA expression of (**D**) IL-1β, (**E**) IL-6 and (**F**) TNF-α. **G** Protein levels of IBA-1 and GFAP. **H** Expression of apoptosis-related proteins Bax and Bcl-2. **I** Expression of BBB junction proteins ZO-1, Occludin, and Claudin-1. **J** Expression of BDNF, PSD95, and Synaptophysin. **K** H&E staining in DG area. Scale bars: 100 μm. **L** Nissl staining in DG area. Scale bars: 100 μm. **M** Golgi staining and density of dendritic spines. Scale bars: 10 μm. **N**-**P** Immunofluorescence for (**N**) IBA-1and GFAP in DG area, (**O**) NeuN and BDNF in CA3 area, (**P**) PSD95 and SYP in CA3 area. Scale bars: 100 μm. CON, normal control. MOD: VaD rats treated with normal saline every other day. LST: VaD rats administered *L. salivarius* every other day. ASP: VaD rats administered ASP every other day. Data are presented as mean ± SEM, *n* = 4 per group. ns. *P* > 0.05, *P < 0.05, **P < 0.01, ***P < 0.001

Next, the effect of intranasal low-dose ASP on the inflammatory response was evaluated in a rat model of VaD. The results showed that serum IL-6, IL-1β and TNF-α concentrations in ASP group were significantly higher than those in LST group, while TNF-α concentrations were lower than those in MOD group (Fig. 5A-C). The mRNA expression of these inflammatory factors in brain tissue of ASP group was also significantly higher than that of LST group, and it was only slightly lower than that of MOD group (Fig. 5D-F, *P* > 0.05). Moreover, the expressions of IBA-1 and GFAP in ASP group were signally lower than those in MOD group, but still higher than those in LST group, indicating that low-dose ASP had a certain inhibitory effect on neuroinflammation, although it was weaker than *L. salivarius* (Fig. 5G). From the perspective of neuroprotection, ASP only induced a slight reversal of the abnormal expression of Bax and Bcl-2, two apoptosis-related proteins, without producing a significant regulatory effect (Fig. 5H). The evaluation of blood-brain barrier function showed that the expression of ZO-1, Occludin and Claudin-1 protein in brain tissue of ASP group was significantly higher than that of MOD group, and there was no significant difference between ASP and LST group (Fig. 5I). Additionally, the protein expressions of BDNF, PSD95 and synaptophysin in ASP group were significantly lower than those in LST group, and only slightly increased compared with MOD group (Fig. 5J, *P* > 0.05). Histological staining in DG region (H&E, Nissl and Golgi staining) showed that ASP intervention reduced the structural damage of brain cells, improved the distribution of Nissl bodies, increased the synaptic density and the number of mature dendritic spines in hippocampus, indicating partial synaptic repair (Fig. 5K-M). Immunofluorescence staining of CA3 and DG regions of the hippocampus further confirmed that ASP reduced the inflammation and injury of nerves to some extent (Fig. 5N-P).

HE staining of lung tissue showed that the alveolar structure of each group was intact and there was no pathological damage (Fig. S7A). Western blot and immunofluorescence results revealed that the expressions of ZO-1, Occludin and Claudin-1 in lung tissue in MOD group were significantly lower than those in CON group, while those in ASP group were signally higher than those in MOD group, and the barrier proteins showed continuous linear distribution (Fig. S7B-H).

These results indicated that while low-dose ASP administered intranasally offers a marked restorative effect on the lung barrier in VaD rats, its benefits for neurological repair are comparatively modest.

### Preparation and in vivo distribution of ASP-Loaded EVs from *L. salivarius*

**Fig. 6:**
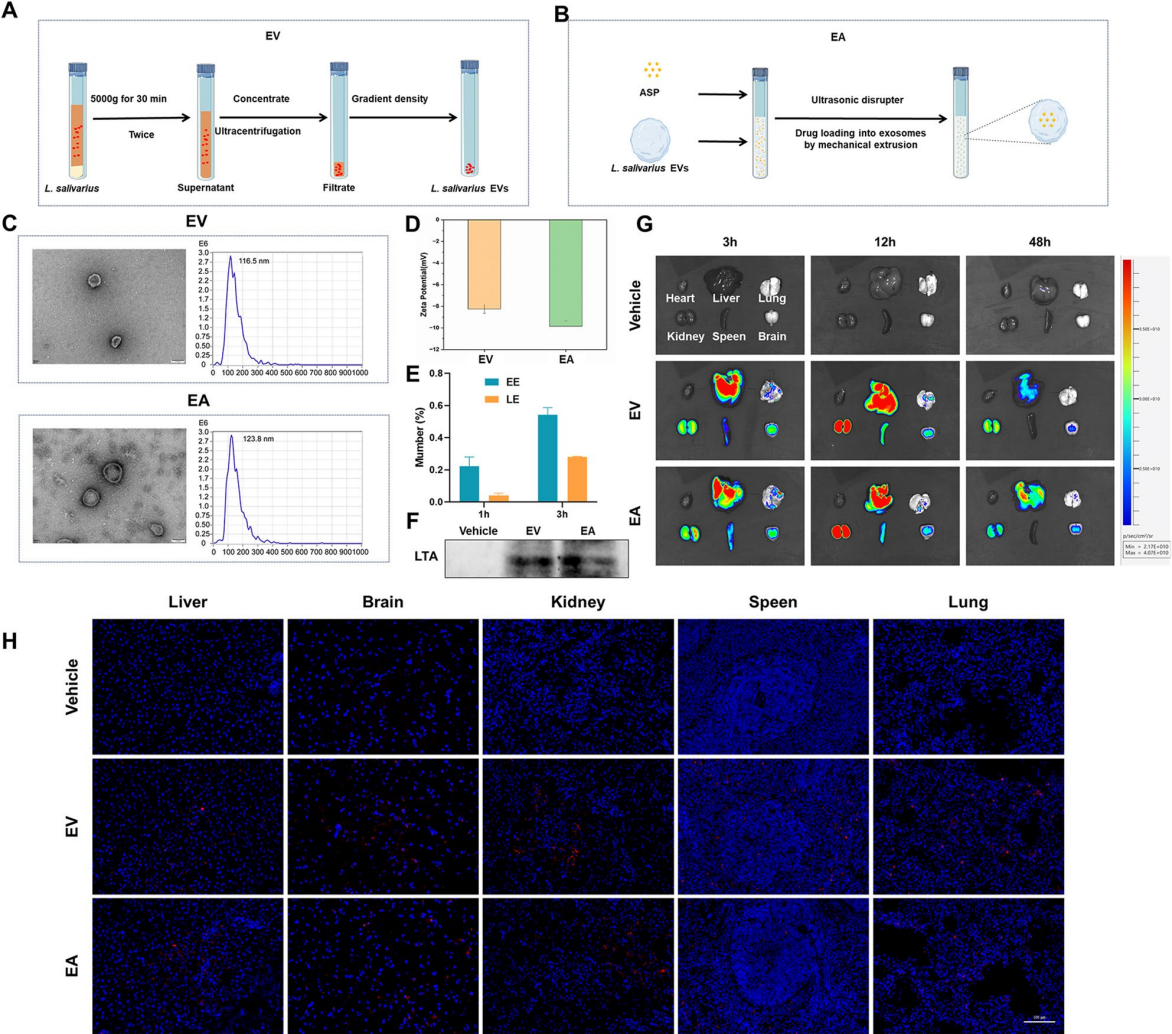
Preparation and in vivo distribution of ASP-loaded extracellular vesicles (EA) derived from *L. salivarius*. **A** Schematic illustration of EV extraction from *L. salivarius*. **B** Schematic procedure of ASP encapsulation into EVs. **C** NTA analysis and TEM images of blank EVs and ASP-loaded EVs (EA). Scale bar: 100 nm. **D** Zeta potential of EV and EA. **E** Encapsulation efficiency and drug loading capacity of EA. **F** Detection of LTA (a Gram-positive bacterial EVs marker) by WB. **G** In vivo fluorescence imaging of major organs at 3, 12 and 48 h after intranasal administration of Dil-labeled EV and EA. **H** Fluorescence images of organ sections (Liver, Spleen, Lung, Kidney, Brain) at 12 h post-administration. Scale bar: 100 μm. *n* = 3 per group

Based on previous study that EVs can cross the blood-brain barrier and accumulate in brain tissue, we propose to wrap EVs in aspartic acid as drug delivery carriers to improve their bioavailability. Figure 6A and B respectively illustrate the extraction process of EVs derived from *L. salivarius* and the schematic procedure of ASP encapsulation. TEM images showed that both blank EVs and drug-loaded vesicles (EA) showed typical vesicle structures (Fig. 6C). NTA revealed that the average diameter of EVs was about 116.5 nm, which increased slightly to 123.8 nm after drug loading (Fig. 6C). Zeta potential measurement showed that the surface charge of EVs is -8.25 mV, while that of EA is-9.84 mV (Fig. 6D). After ultrasonic treatment for 30 min and incubation for 3 h, the encapsulation efficiency of EA reached 54.22% and the drug loading was 28.14% (Fig. 6E). Next, we identified the characteristic protein LTA of EVs and EVS-Asp and demonstrated that both are outer membrane vesicles of Gram-positive bacteria (Fig. 6F). Subsequently, we systematically evaluated the distribution of EVs and EA in the main organs (heart, liver, spleen, lung, kidney and brain) of rats after intranasal administration using in vivo imaging (Fig. 6G). The findings indicated that ASP encapsulation did not alter the tissue distribution profile of EVs. At 3 h post-administration, a prominent fluorescence signal was evident in lung tissue, with the most intense signals observed in the liver and kidney. Additionally, noticeable signals were present in the spleen and brain. By the 12-hour mark, the lung signal had diminished, whereas signals in the kidney and spleen had intensified. The signals in the liver and brain exhibited stability. Notably, even at 48 h, when the lung signal had nearly dissipated, a substantial retention of fluorescence was still discernible in brain tissue. Moreover, frozen tissue sections obtained 12 h post-administration directly verified the distribution of EVs and EAs in different target organs (Fig. 6H). Notably, these results reaffirmed that EVs could cross the blood-brain barrier, enter brain tissue, and accumulate therein. These findings indicated that EVs derived from *L. salivarius*, as a delivery carrier of ASP, can transport drugs to brain tissue while retaining its inherent targeting characteristics.

### EVs-ASP can improve cognitive dysfunction and promote nerve and barrier repair in VaD rats

**Fig. 7:**
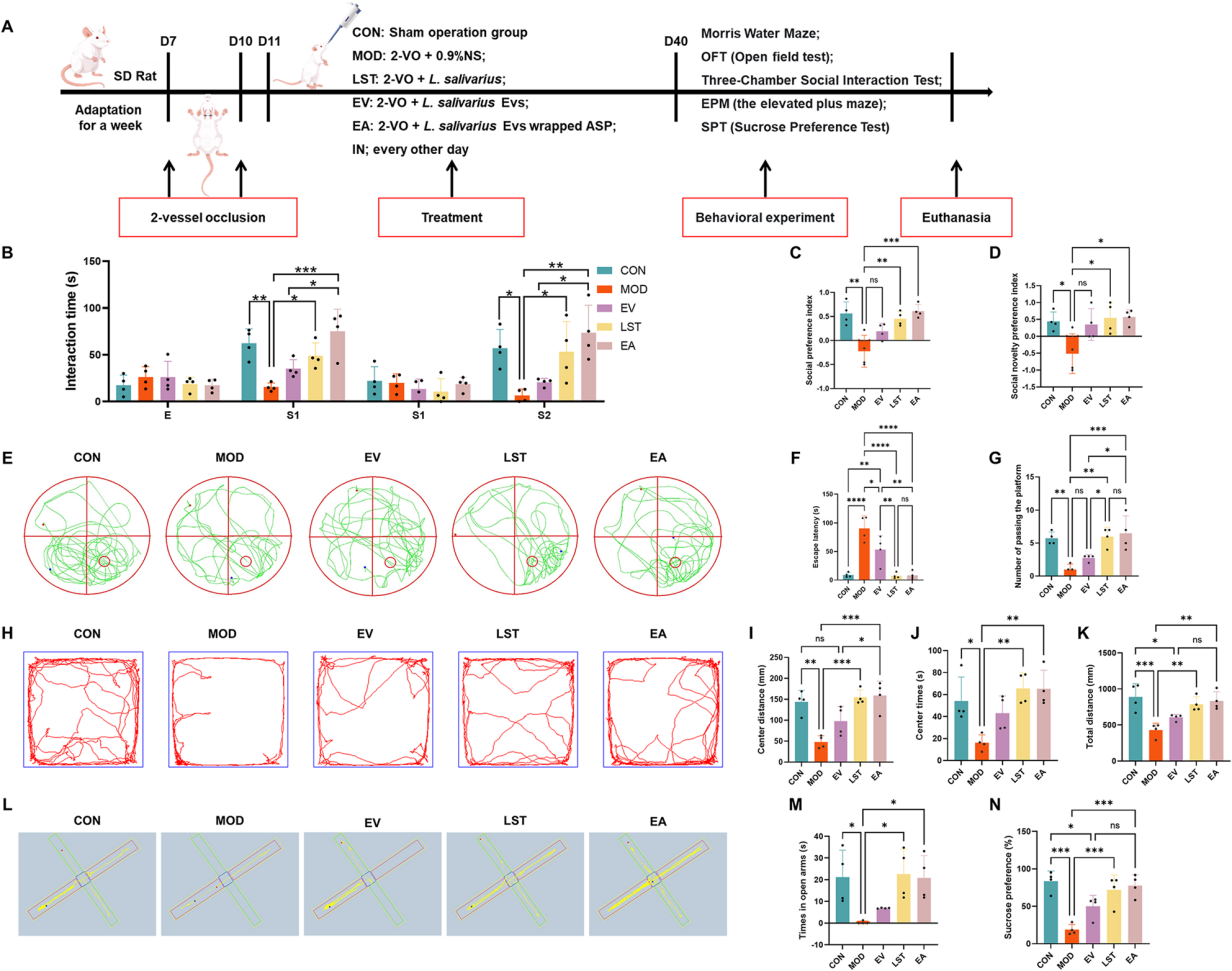
EA improves cognitive and behavioral functions in VaD rats. **A** Experimental timeline. **B**-**D** Three-chamber social test: (**B**) time spent in the three chambers, (**C**) social preference index, and (**D**) social novelty index. **E**-**G** Morris water maze: (**E**) representative paths, (**F**) escape latency, and (**G**) platform crossings. **H**-**K** Open field test: (**H**) representative movement trajectories, (**I**) total distance, (**J**) center distance, (**K**) center time. **L**, **M** Elevated plus maze: (**L**) Representative movement trajectories of elevated plus maze, and (**M**) open arm time. **N** Sucrose preference ratio. CON, normal control. MOD: VaD rats treated with normal saline every other day. EV: VaD rats administered EVs every other day. LST: VaD rats administered *L. salivarius* every other day. EA: VaD rats administered EAs every other day. Data are presented as mean ± SEM, *n* = 4 per group. ns. *P* > 0.05, *P < 0.05, **P < 0.01, ***P < 0.001

To explore the effects of EA on cognitive function and many physiological indexes of VaD rats, EV group (simple extracellular vesicle group) and EA group (ASP-loaded vesicle group) were added to the original experiment, and compared with control group (CON), model group (MOD) and *L. salivarius* group (LST) systematically (Fig. 7A). The results of behavioral experiments showed that EA intervention can significantly improve the cognitive dysfunction of VaD rats, and its effect was equivalent to that of LST group, which was close to the level of CON group and better than that of EV group. In various tests including the Three-Chamber social interaction (Fig. 7B-D; *P* < 0.05), water maze (Fig. 7E-G; *P* < 0.001), open field (Fig. 7H-K; *P* < 0.01), plus maze (Fig. 7L, M; *P* < 0.05), and sucrose preference (Fig. 7N; *P* < 0.001), the EA group exhibited significant improvements in social preference, spatial memory, exploratory behavior, anxiety-like behavior, and anhedonia compared to the MOD group. These improvements were comparable to those of the LST group, while the EV group showed some enhancement, albeit to a lesser extent than the EA group.

**Fig. 8:**
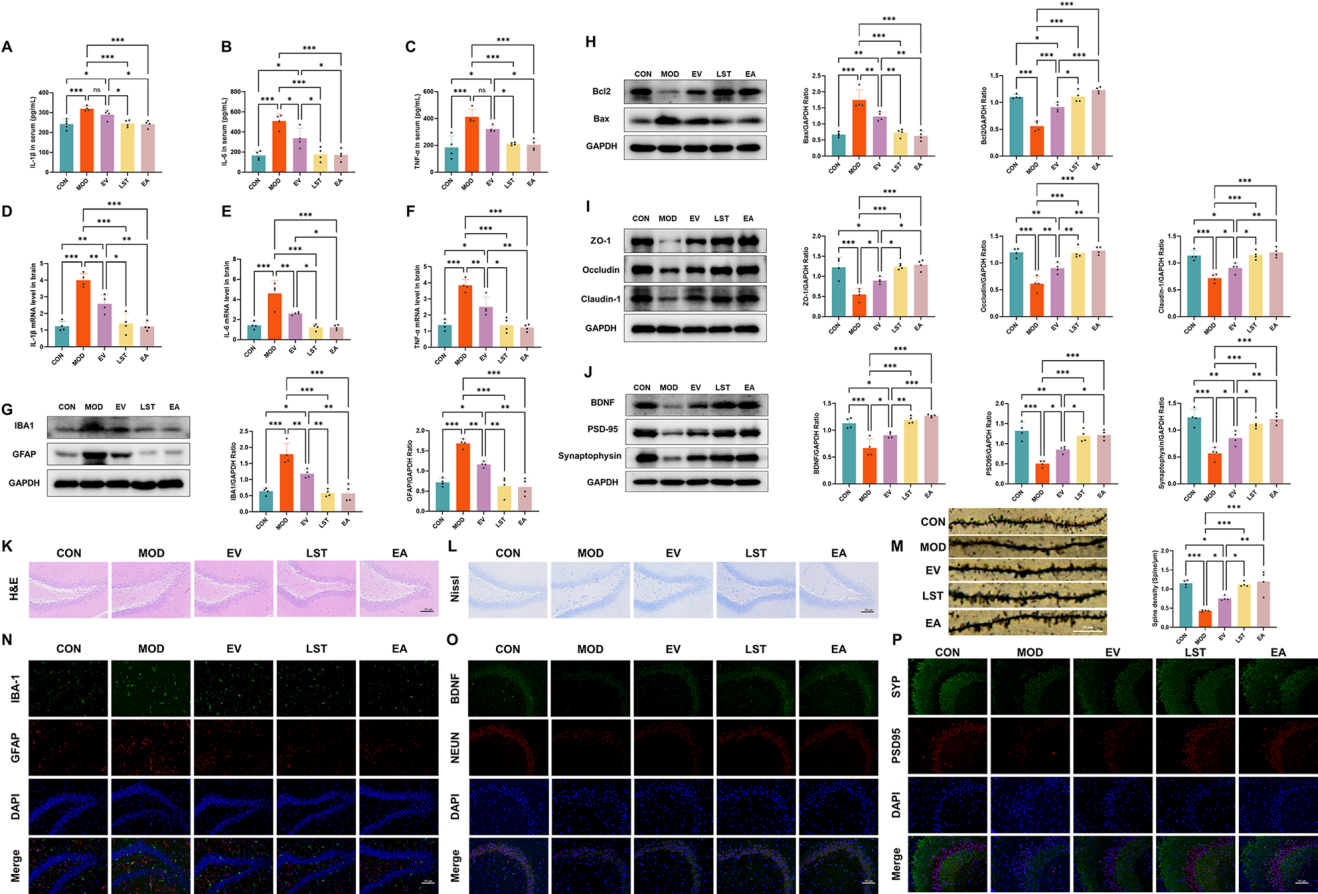
EA attenuates neuroinflammation, protects BBB integrity, and promotes neural repair. **A**-**C** Serum levels of (**A**) IL-1β, (**B**) IL-6, and (**C**) TNF-α. **D**-**F** Brain mRNA expression of IL-1β (**D**), IL-6 (**E**), and TNF-α (**F**). **G** Protein levels of IBA-1 and GFAP. **H** Expression of Bax and Bcl-2. **I** Expression of tight junction proteins ZO-1, Occludin, and Claudin-1. **J** Expression of BDNF, PSD95, and Synaptophysin. **K**-**M** Histological staining: (**K**) H&E in DG area, (**L**) Nissl in DG area and (**M**) Golgi and density of dendritic spines. Scale bars: 100 μm (**K **and **L**), 10 μm (**M**). **N**-**P** Immunofluorescence for (**N**) IBA-1and GFAP in DG area, (**O**) NeuN and BDNF in CA3 area, (**P**) PSD95 and SYP in CA3 area. Scale bars: 100 μm. CON, normal control. MOD: VaD rats treated with normal saline every other day. EV: VaD rats administered EVs every other day. LST: VaD rats administered *L. salivarius* every other day. EA: VaD rats administered EAs every other day. Data are presented as mean ± SEM, *n* = 4 per group. ns. *P* > 0.05, *P < 0.05, **P < 0.01, ***P < 0.001

EA had similar protective effect to LST in inflammatory reaction, apoptosis and blood-brain barrier function. The results of ELISA, qRT-PCR and Western blot showed that EA could significantly reduce the levels of inflammatory factors such as IL-6, IL-1β and TNF-α in serum and brain tissue (Fig. 8A-F), inhibit the overactivation of microglia and astrocytes (Fig. 8G), regulate the expression of Bax/Bcl-2 protein to inhibit apoptosis (Fig. 8H), and effectively up-regulate the expression of tight junction proteins (ZO-1, Occludin, Claudin-1) (Fig. 8I). Among the above indexes, the improvement degree of EA group is equivalent to that of LST group, which is sharply better than that of MOD group and EV group.

In nerve repair, EA intervention can markedly up-regulate the protein expressions of BDNF, PSD95 and Synaptophysin in brain tissue, promote the reconstruction of synaptic structure, and increase the density and mature ratio of dendritic spines in hippocampus (Fig. 8J). The results of tissue staining (H&E, Nissl and Golgi staining) and immunofluorescence in CA3 and DG regions of the hippocampus showed that EA treatment could effectively alleviate nerve cell injury, improve Nissl distribution and synaptic morphology, and its repair effect was similar to that of LST group, significantly better than that of EV group, and close to that of CON group (Fig. 8K-P). Similarly, nasal administration of EA and EV did not cause pathological changes in the lungs (Fig. S8A). In addition, the detection of lung tissue barrier function showed that EA treatment could significantly restore the expression of ZO-1, Occludin and Claudin-1 in lung tissue, improve the distribution of tight junction protein, and its repair effect was equivalent to that of LST group, and no obvious pathological damage was observed in alveolar structure of each group (Fig. S8B-H).

In conclusion, intranasal EA administration effectively ameliorated the pathological condition of VaD rats across various domains including behavior, inflammation suppression, nerve regeneration, and barrier fortification. The overall therapeutic impact is comparable to that of *L. salivarius*, markedly superior to the simple EV cohort, and approaches that of the normal control group.

### Regulation of *L. salivarius* on gene expression profile in brain tissue of VaD rats

**Fig. 9:**
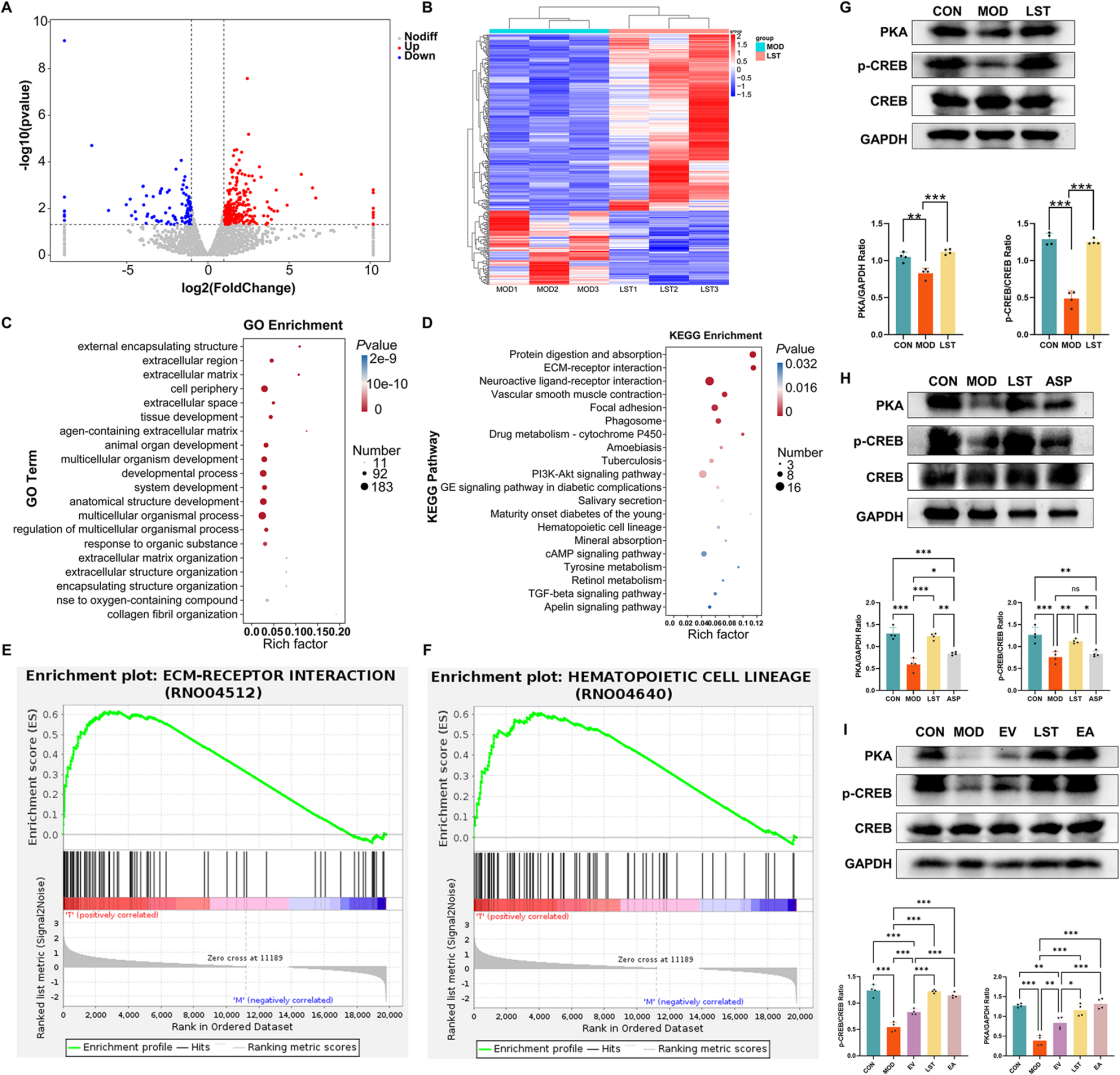
RNA-Seq of brain tissues reveals regulatory mechanisms of *L. salivarius* in VaD rats. **A** Volcano plot of differentially expressed genes (DEGs) between the MOD and LST groups. Significantly up- and down-regulated genes are highlighted in red and blue, respectively, based on the thresholds of |log₂FC| > 1 and FDR < 0.05. **B** Heatmap of DEG expression between MOD and LST groups. Rows represent genes, columns represent samples, with red and blue indicating high and low expression levels, respectively. **C** GO enrichment analysis of DEGs. **D** KEGG pathway enrichment analysis of DEGs. **E**, **F** The GSEA pathway enrichment analysis of the ECM-receptor interaction and Hematopoietic cell lineage. **G**-**I** Expression of PKA, p-CREB and CREB(*n* = 4 per group). MOD: VaD rats treated with normal saline every other day. LST: VaD rats administered *L. salivarius* every other day. *n*= 3 per group

To further study the regulation mechanism of *L. salivarius* on gene expression pattern in VaD rats, RNA sequencing (RNA-Seq) was performed on brain tissue samples from model group (MOD) and *L. salivarius* treatment group (LST). Systematic comparison was made to determine the difference of gene expression profile between the two groups. RNA-Seq analysis revealed significant differences in gene expression between MOD group and LST group. A total of 338 differentially expressed genes (DEGs) were identified from the co-expression gene pool, of which 238 were significantly up-regulated and 100 were significantly down-regulated (Fig. 9A, B). To clarify the function and biological significance of these deg, the enrichment analysis of gene ontology (GO) and Kyoto Encyclopedia of Genes and Genomes (KEGG) were carried out. GO enrichment analysis shows that deg is mainly related to biological processes and cell components, such as “cell periphery”, “developmental process” and “multicellular biological process”, which indicates that these genes may play an important role in maintaining cell structure, regulating developmental process and regulating the overall physiological function of brain tissue (Fig. 9C). KEGG pathway analysis showed that the DEGs were mainly enriched in pathways including “protein digestion and absorption,” “ECM-receptor interaction,” and “neuroactive ligand-receptor interaction” (Fig. 9D). These pathways are closely associated with nutrient metabolism, cell adhesion, and neural signaling, respectively—all of which represent key regulatory mechanisms in the pathogenesis of VaD. In addition, gene set enrichment analysis (GSEA) confirmed that compared with MOD group, the brain tissue samples in LST group were significantly enriched in “ECM- receptor interaction” and “hematopoietic cell lineage” (Fig. 9E, F). Briefly, the RNA-Seq study showed that *L. salivarius* significantly regulated the gene expression profile in the brain tissue of VaD rats. The regulated genes and enrichment pathways are closely related to the maintenance of nerve function and the repair of pathological injury, which indicates that this transcription regulation may represent the key molecular mechanism of *L. salivarius* to alleviate the pathological symptoms of VaD rats.

In this study, we further investigated the role of cAMP/PKA/CREB pathway in this process. In VaD rats, the protein expression of PKA and p-CREB decreased significantly, while the protein expression of LST, EVs and EVS-Asp treatment was higher than that of MOD group (Fig. 9G-I). These results suggest that the improved cognitive dysfunction in VaD rats may be related to this pathway.

## Discussion

Vascular dementia (VaD) is the second most common type of dementia in the world, which involves complex pathophysiology and is characterized by the interaction of multiple processes, such as abnormal cerebral blood flow, neuroinflammation and the destruction of blood-brain barrier [34]. Clinical treatment has long been limited by single target intervention. In addition, there are various animal models for VaD, While the 2‑VO model is a classic rodent model of chronic cerebral hypoperfusion for vascular dementia. Although this model has certain limitations, it can reliably reproduce diffuse white matter lesions, which are consistent with the pathological features of human vascular dementia. White matter is inherently vulnerable to hypoperfusion across species, and chronic global hypoperfusion models such as 2‑VO can mimic ischemic white matter injury similar to that in patients. Thus, the 2‑VO model shows acceptable translational value for studying hypoperfusion‑related vascular dementia. The traditional research on VaD mainly focuses on the “gut-brain axis” regulatory network, and it is believed that the ecological imbalance of intestinal microflora affects the central nervous system through metabolites (such as SCFAs and trimethylamine N- oxides) or immune signals [35, 36]. However, lung, as the largest mucosal immune organ in human body, has long been neglected because of its microbial correlation with the function of central nervous system [37]. In a study on the lung microbiome and multiple sclerosis, neomycin treatment increased the composition of the lung microbiome, resulting in bacteria producing more LPS, which crossed the blood-brain barrier and entered the central nervous system, promoting IFN production in type I microglia, thus playing a neuroprotective role [38]. Some studies have found that the brain tissue after severe acute pneumonia has a flora similar to that of the lung, suggesting that there is some link between the lung and the brain, but we still do not know. In this study, we systematically studied the “*L. salivarius*-lung-brain axis” and developed a comprehensive strategy combining “microbial intervention, metabolite targeting and nano-delivery” through three-stage experimental design. This method offers a novel approach to cross-organ regulation for VaD treatment and contributes to elucidating the connection between lung microecology and central nervous system pathology through comprehensive mechanism analysis.

Studies have shown that lung microflora can continuously communicate with microglia (immune cells of the brain) and regulate their immune reactivity [39]. Moreover, considering the direct exposure of the lung to the external environment, its microbial flora could potentially function as a distant early alert system for the central nervous system. This study confirmed the VaD animal model by demonstrating that the model rats exhibited neuroinflammation and cognitive deficits. Additionally, these rats showed an imbalance in lung microecology along with impaired lung barrier function. Previous study has pointed out that neomycin can reduce central inflammation and change autoimmune response in autoimmune encephalomyelitis model by affecting the function of microglia in the central nervous system of rats, suggesting that the changes of pulmonary microbiota are related to the autoimmune process in the brain by regulating the central microglia [6]. However, after nasal administration of *L. salivarius*, the lung flora structure of rats recovered (the abundance of *Lactobacillus* increased), the lung barrier integrity was rebuilt, and the central nervous inflammation was observably relieved and the cognitive function was improved. When exploring the neurological pathogenesis associated with SARS-CoV-2 infection, it has been shown that systemic inflammation may aggravate neurological complications. Another study showed that certain molecules in the lung can limit brain damage after systemic inflammatory activation [40], so our study suggests that VaD may cause systemic inflammation and aggravate cognitive dysfunction vascular dementia nction in rats, while *L. salivary* and EVs-ASP supplementation may be beneficial factors in inhibiting neuroinflammation. The synchronization of this “lung-brain” pathological relief confirmed that the lung microenvironment was the key to the pathological progress of VaD, and supported the core regulatory position of “microbiota-lung-brain axis” in VaD, providing a clear target direction for cross-organ intervention.

The complexity of the pathological mechanism of VaD determines that it is difficult to achieve the ideal effect by single-target intervention. This research revealed that the neuroprotective impact of *L. salivarius* is not contingent on a singular pathway but rather results from a synergistic effect involving multiple immune-metabolic-neural systems. This finding aligns well with the complex clinical manifestations of VaD, characterized by multifactorial etiology and interconnected pathways. *L. salivarius* exhibits dual anti-inflammatory effects by modulating the immune response. Specifically, it can concurrently reduce serum levels of IL-6 and IL-1β, consistent with findings reported by *Wang et al.* (2024) on the ability of *L. salivarius* (SA-03) to decrease inflammation markers [38] such as serum IgE, IL-5, and the proportion of CD4 + cells in the lungs [41]. It is worth noting that *L. salivarius* can reduce intestinal leakage and oxidative stress by antagonizing the C/EBPβ/AEP signal axis in the intestine, thus affecting AD-related diseases, suggesting that the “peripheral-central” anti-inflammatory mode of probiotics may be a common mechanism across dementia types, which provides support for the universality of the results of this study [42]. Notably, intranasal *L. salivarius* synchronously upregulated the tight junction proteins Occludin, Claudin-1 and ZO-1, and restored both lung barrier and BBB integrity in VaD rats. We propose two potential mechanistic pathways underlying this dual reparative effect. The first is parallel independent direct regulation, where *L. salivarius* directly repairs the lung barrier via respiratory mucosal colonization, while its secreted EVs and metabolite ASP exert direct BBB-protective effects independent of lung repair [8, 43]. The second is causal cascade regulation, where lung barrier repair acts as the upstream driver of BBB protection. Lung barrier damage triggers systemic inflammation as a core mediator of VaD-related BBB injury [44], and our data support that *L. salivarius* may indirectly protect the BBB by repairing the lung barrier to suppress systemic inflammation, with a strong correlation observed between its anti-inflammatory effect and improved BBB integrity. Admittedly, the independent contribution of these two mechanisms remains to be fully delineated, and follow-up studies will verify their causal relationship to advance understanding of the microbiota-lung-brain axis.

At the level of metabolic regulation, un-targeted metabolomic analysis revealed that *L. salivarius* can concurrently modulate the metabolic profiles of both lung and brain tissues. Among the altered metabolites, the identification of the core active metabolite ASP—an iridoid glycoside—proved to be of particular significance. Previous studies have reported that ASP possesses antioxidant and anti-inflammatory properties; however, its specific role in VaD remains unclear [45]. Previous study has shown that ASP can alleviate acute lung injury by inhibiting NF-κB translocation and MAPK phosphorylation [20]. At a dosage of 40 mg/kg, O-glycosylation promotes IκBα while inhibiting its ubiquitination and phosphorylation. This process blocks the activation of the NF-κB signaling pathway, thereby reducing apoptosis and injury in hippocampal neurons. Consequently, this intervention ameliorates depression-like behavior and cognitive impairment induced by chronic stress. In addition, the dose of ASP used for intraperitoneal injection was 20–80 mg/kg [46], and the oral dose was 20–40 mg/kg [47]. Notably, a low oral dose range of 0.125–0.5 mg/kg has been reported to exert significant therapeutic effects in chronic colitis [48]. The absorption efficiency and bioavailability of intranasal administration are similar to those of intravenous administration, and both are significantly higher than those of oral administration. Therefore, a much lower dose can be used for intranasal administration compared with oral or intraperitoneal administration. Based on the above literature reports and the high bioavailability of intranasal administration, we selected 2 mg/kg as a low but effective dose in the present study to achieve satisfactory pharmacological effects while minimizing potential systemic exposure. These findings offer a novel therapeutic approach for nervous system disorders, particularly depression.

These findings offer a novel therapeutic approach for nervous system disorders, particularly depression [21]. This study further found that low-dose ASP (2 mg/kg) alone has limited neuroprotective effect, but it can specifically repair the integrity of lung barrier. This tissue-specific effect may be related to its dose dependence and lack of organ targeting, suggesting that the precise combination of “metabolites-organ receptors” may be the key mechanism in the metabolic regulation of “microbiota-lung-brain axis”. At the level of nerve repair, *L. salivarius* treatment can up-regulate the expression of neurotrophic factors (such as BDNF) and promote protein synthesis and synaptic reconstruction of neurons [49]. Previous studies have shown that Tongqiao-Huoxue decoction can promote synaptic remodeling in rats with vascular dementia through the cAMP/PKA/CREB pathway [50]. It is well known that phosphorylation of the cAMP/PKA/CREB pathway is a key upstream mechanism mediating transcriptional activation of BDNF. As an important intracellular second messenger, cAMP activates camp-dependent protein kinase A (PKA), which phosphorylates the transcription factor CREB, promotes gene transcription, and enhances the synthesis of the downstream protein BDNF. However, the increase of BDNF inhibited neuronal apoptosis and improved cognitive dysfunction. At the same time, the activation of “extracellular matrix-receptor interaction” pathway indicates that *L. salivarius* may maintain synaptic stability and neuronal homeostasis by regulating the composition and structure of extracellular matrix, a mechanism that has received little attention in VaD research [51]. In addition, the enrichment of the “hematopoietic cell lineage” pathway suggests that the strain may indirectly inhibit neuroinflammation by regulating the function of immune cells, and further improve the “lung-immune-brain” axis [52].

The clinical transformation of natural active metabolites has long been hindered by its poor stability and low brain targeting efficiency [53, 54]. To meet this challenge, a drug delivery system based on EVs of *L. salivarius* was developed in this study. By integrating “carrier therapy” and “drug therapy”, this synergistic strategy effectively overcomes the above limitations. Compared with mammalian EVs, bacterial EVs (BEVs) have the advantages of short culture period, strong expansibility and rich bioactive molecules [55]. BEVs can cross the blood-brain barrier, regulate immune response, promote tissue repair and effectively deliver therapeutic agents. These characteristics emphasize their powerful therapeutic potential for central nervous system diseases such as ischemic stroke [56]. Study has shown that EVs from *L. paracasei* can reduce neuroinflammation and improve cognitive function in hyperammonemia rat model [57]. Our previous work confirmed that oral *L. salivarius* EVs can cross the blood-brain barrier and accumulate in brain tissue [31]. This study further proved that even when loading the exogenous compound ASP, *L. salivarius*-EVs still maintained its ability to effectively penetrate the blood-brain barrier, while maintaining its targeting specificity. This indicated that the surface biomarkers on EVs may mediate the process of “endothelial cell receptor recognition” and provide molecular basis for accurate drug delivery. Mechanistically, previous study has proved that exosomes from *L. reuteri* EVs can bind with Toll-like receptor 2 (TLR2) on brain microvascular endothelial cells (such as bEnd.3 cells) through peptidoglycan (PGN) carried by them, thus mediating intercellular transport and promoting blood-brain barrier crossing [56]. The efficacy of the EA drug delivery system surpasses that of free ASP, underscoring the critical need for optimizing pharmacokinetics in drug delivery platforms. Furthermore, our study demonstrates that even at low doses, the EA system achieves comparable therapeutic effects to *L. salivarius*. This approach not only mitigates potential drug-related toxicity and adverse effects but also aligns with clinical requirements for low-dose, high-efficiency treatment modalities.

This study has certain limitations, mainly including the following aspects: Firstly, the source mechanism of the core active metabolite ASP has not been clarified. Existing evidence only confirms that the content of ASP in the body increases after *L. salivarius* intervention and is correlated with pathological improvement, but direct verification evidence is lacking (such as the detection of ASP in in vitro cultures of *L. salivarius*). Moreover, the possibilities that ASP originates from the dietary intake of rats, the transformation of endogenous precursor substances in the host, or the biotransformation of precursors by the strain have not been ruled out, and no verification of related precursor substances and transforming enzymes has been conducted. Secondly, the bilateral common carotid artery ligation model used here mainly simulates VaD under the condition of chronic cerebral hypoperfusion. Therefore, the treatment strategy needs to be further verified in VaD models with different pathological mechanisms. Thirdly, the precise mechanism of the direct signaling pathway linking the lung microbiota to the central nervous system remains unclear. Specifically, the impact of microbial metabolites on brain function through circulatory or neurological routes requires elucidation through experimental approaches, including microbial colony transplantation and metabolite infusion. Fourthly, no positive drugs were set as controls in this study, which may limit the comprehensiveness of evaluating the therapeutic effect of the intervention. In addition, the mechanism by which EV-ASP (EA) exerts its function remains unclear. Furthermore, whether ASP can enhance the neuroprotective effects of EVs, and whether it acts synergistically with EVs or is delivered by EVs into brain tissue to mediate its biological effects, still requires further experimental validation. Finally, the mass production and quality control system of electric vehicle drug delivery platforms remains inadequately developed. Enhancing the isolation efficiency of EVs and ensuring batch-to-batch consistency are crucial challenges that need resolution to advance clinical translation.

**Fig. 10:**
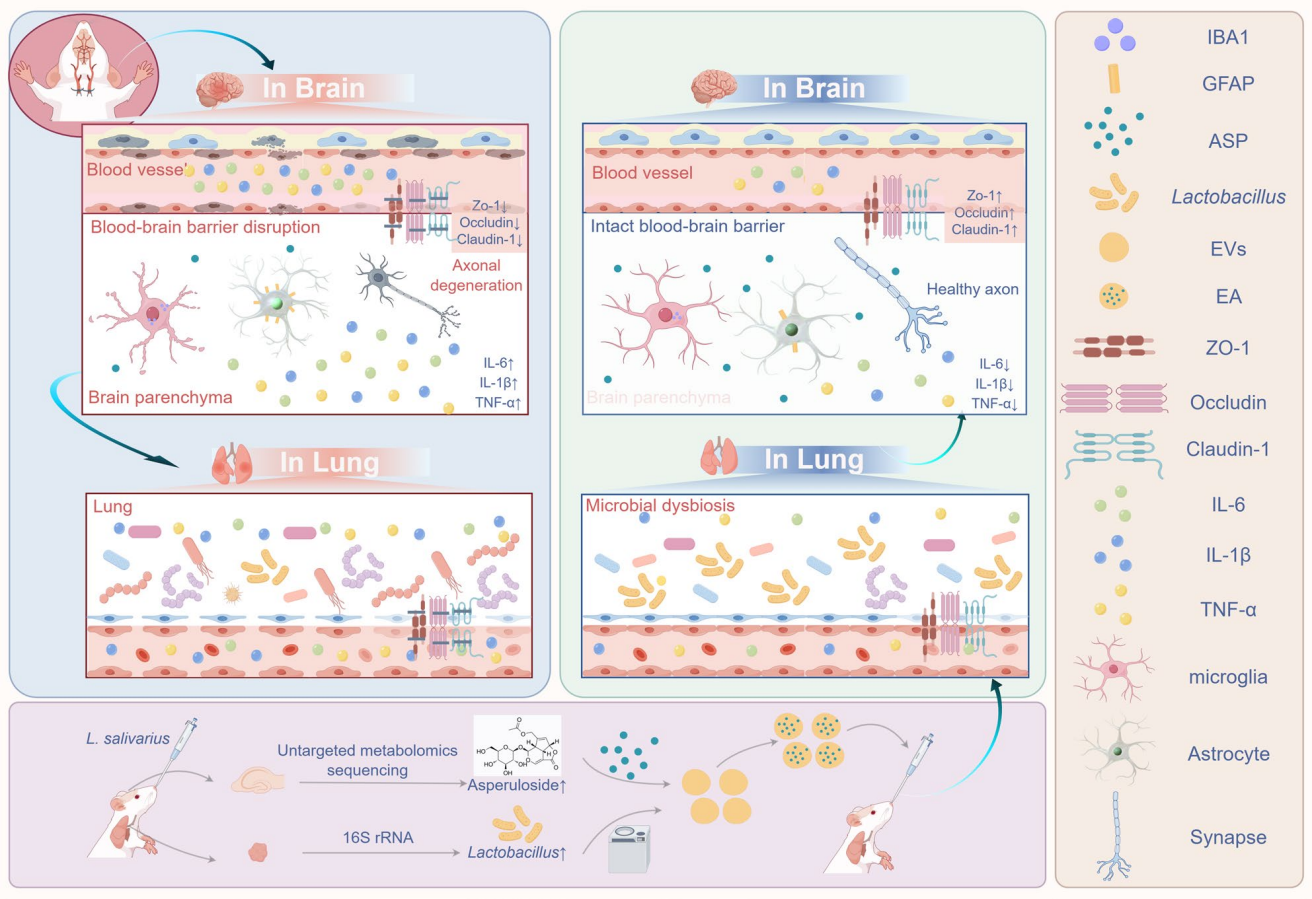
A schematic diagram depicting the proposed mechanism and key findings of this study

In summary, this study adopts multidimensional experimental method, which proves for the first time that *L. salivarius* regulates the pathological progress of VaD through “microbiota-lung-brain axis”, and expounds the potential mechanism from the perspectives of inflammation, metabolism and neurology. In addition, a drug delivery system based on BEVs was successfully developed, which effectively solved the key clinical translation challenges of natural active metabolites, such as poor stability and low targeting efficiency. This study makes three key contributions. Firstly, it enhances the comprehension of VaD pathogenesis by introducing the concept of the “lung–brain axis,” thereby expanding the significance of lung microecology in VaD investigations. Secondly, it establishes an integrated intervention strategy utilizing “nano-carriers of microbial metabolites,” providing a new systematic method for addressing intricate diseases. Lastly, it validates the viability of employing EVs from probiotics as a drug delivery system, thereby establishing a technical basis for precise therapy of central nervous system disorders (Fig. 10). Continuous advancements in validation methods, comprehensive analysis of signaling pathways, and enhancements in drug-loading techniques have paved the way for the potential of the “*L. salivarius*-lung-brain axis” regulation approach as a novel therapeutic pathway for VaD treatment. Furthermore, this strategy could offer significant implications for interventional research on various neurodegenerative disorders, including Alzheimer’s and Parkinson’s diseases.

## Supplementary Information

Supplementary Material 1.

Supplementary Material 2.

## Data Availability

All data supporting the findings of this study are available within the paper and its Supplementary Information.
