# Supplementary figures and images for: The lung-brain axis mediates the neuroprotective effects of nasally administered L. salivarius and its EV-delivered metabolite in vascular dementia
Source: J Neuroinflammation. 2026 May 12;23:235. doi: 10.1186/s12974-026-03864-6 (PMC13343794; doi:10.1186/s12974-026-03864-6)

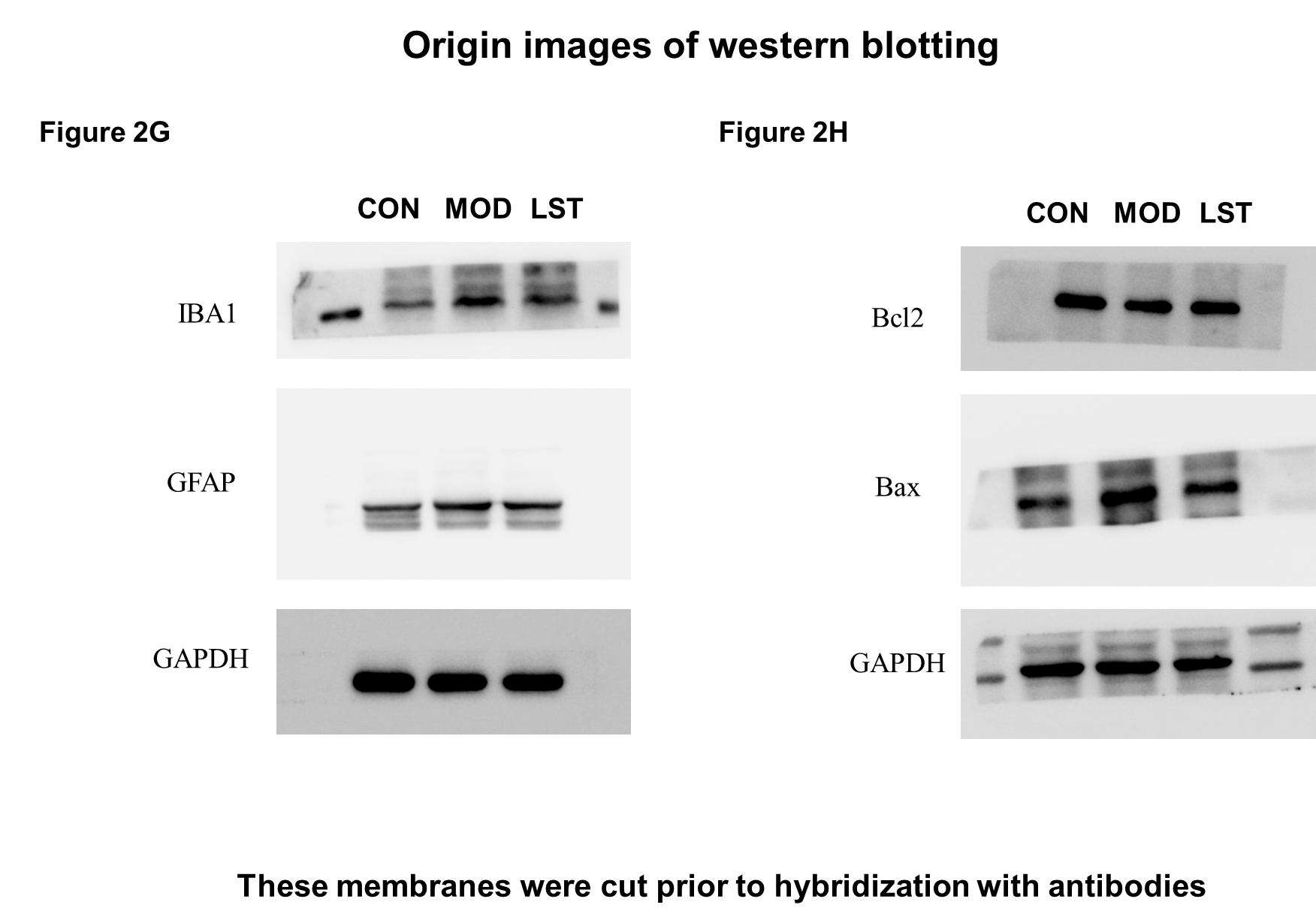


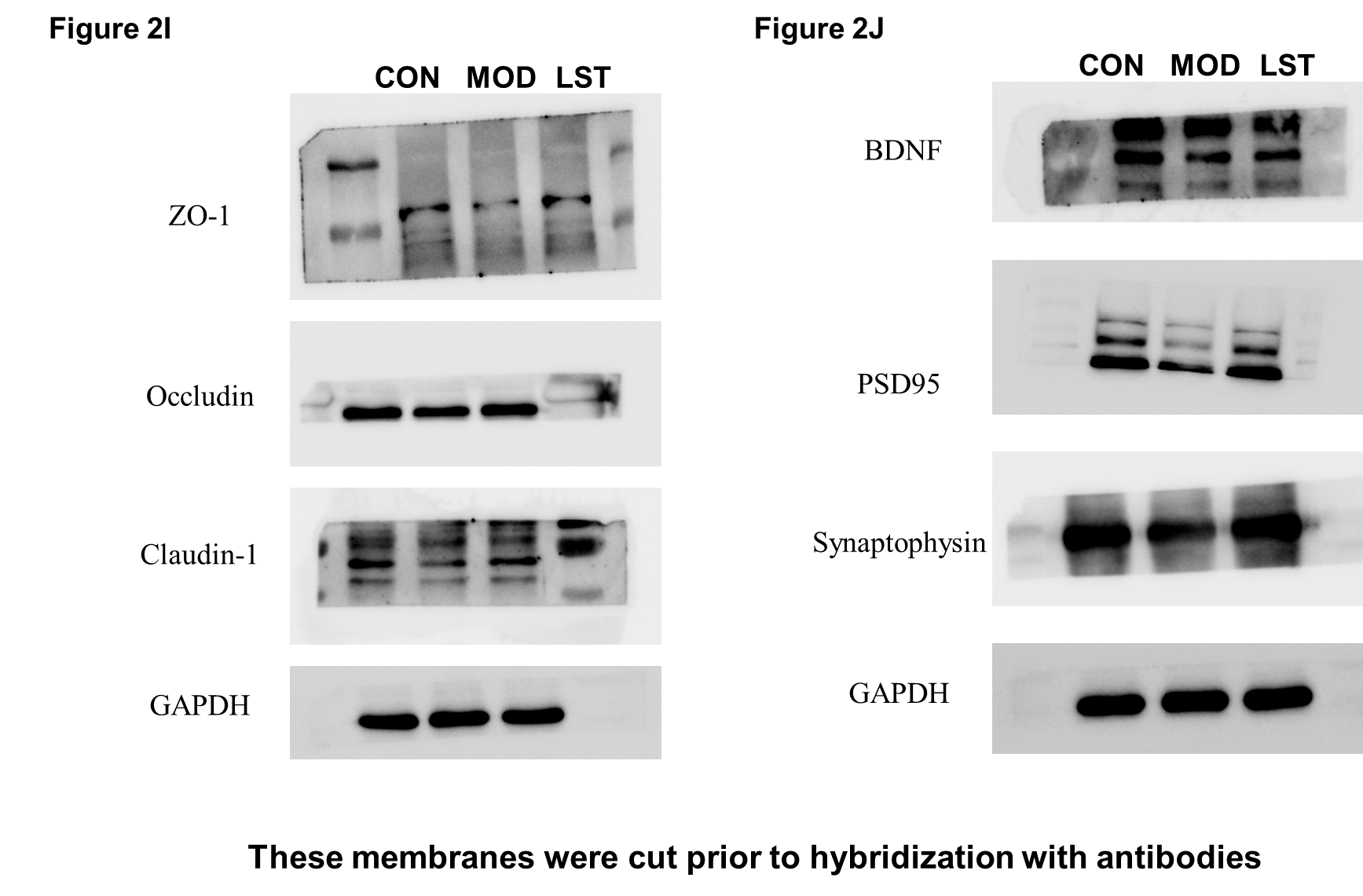


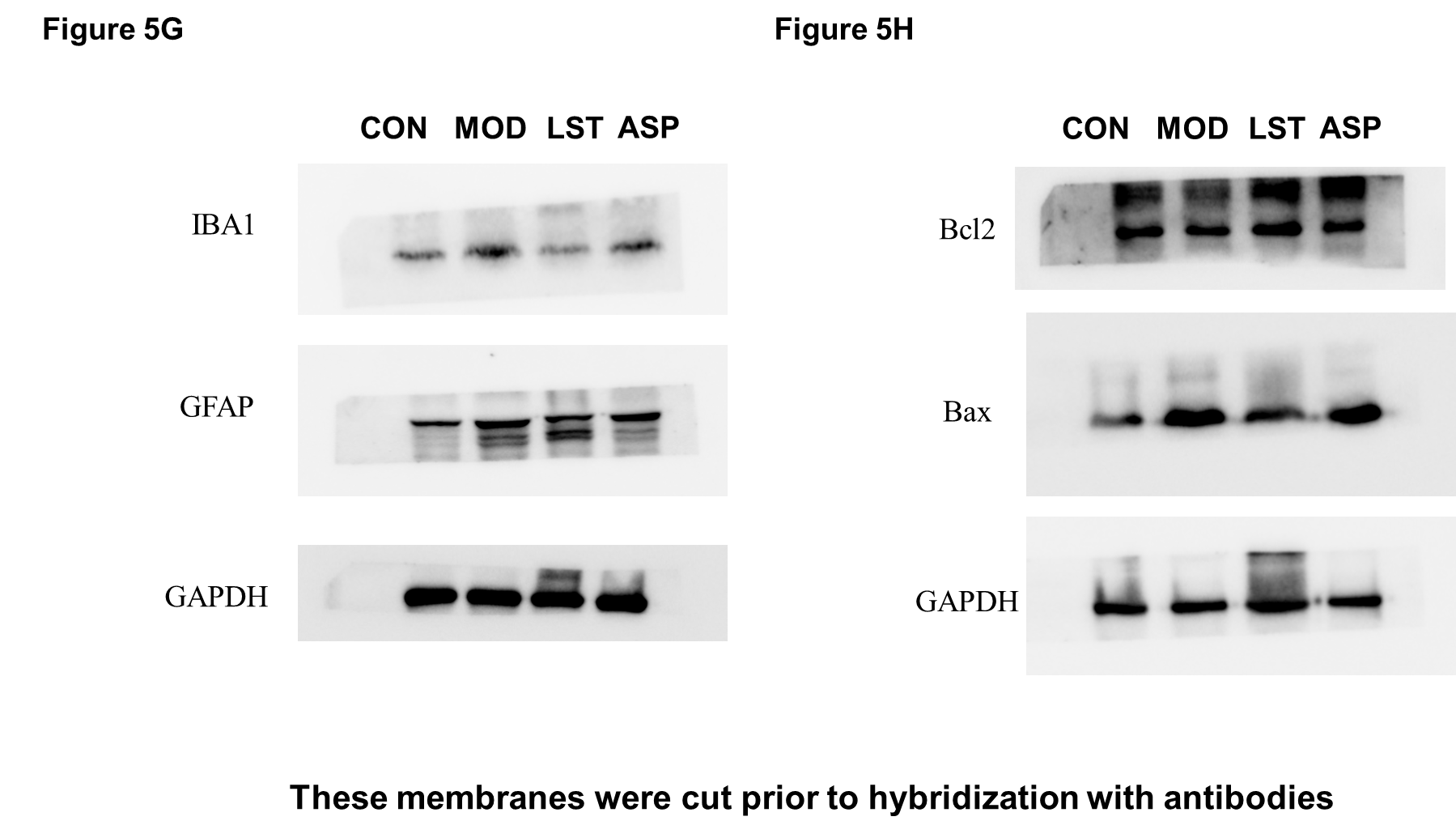


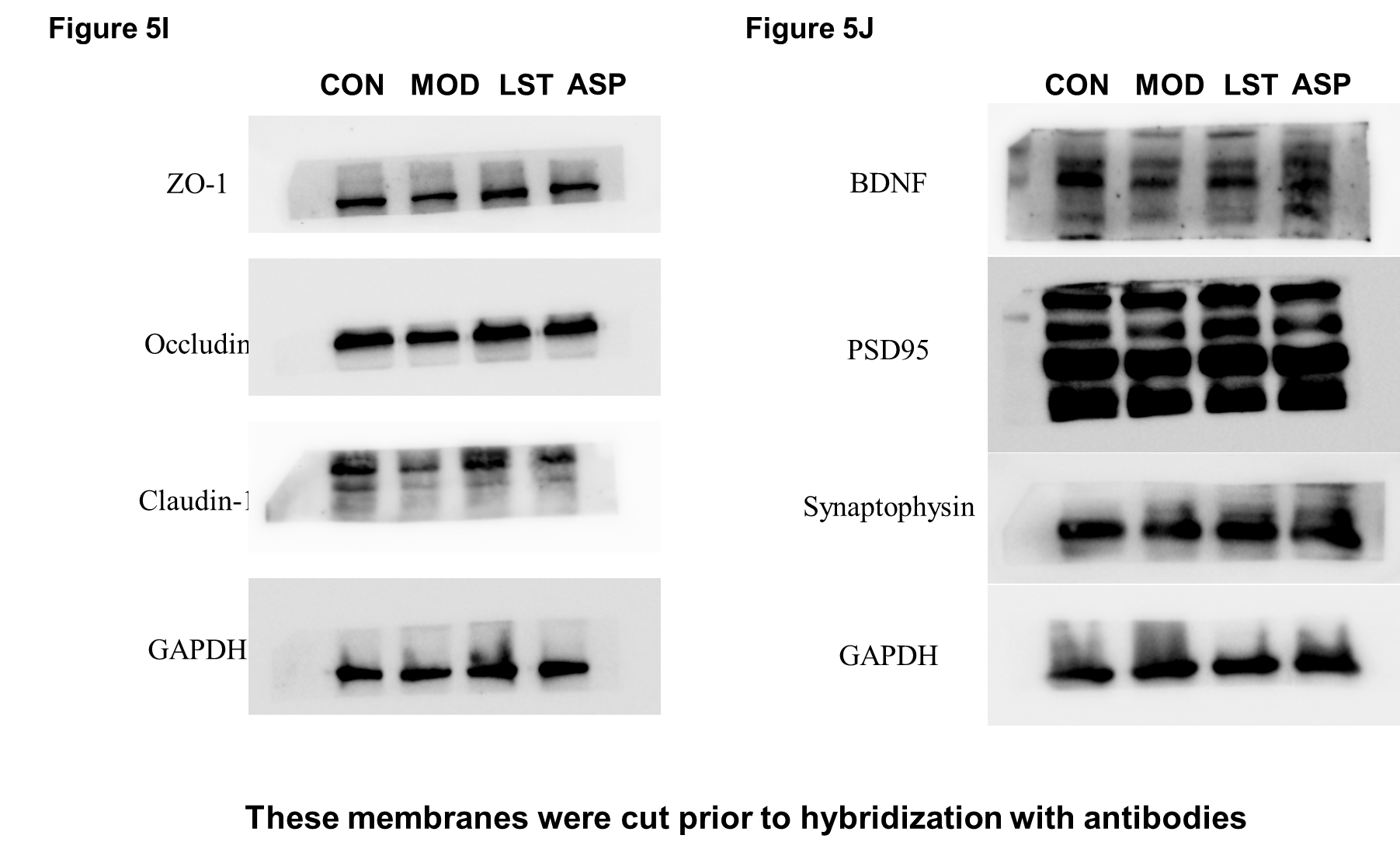


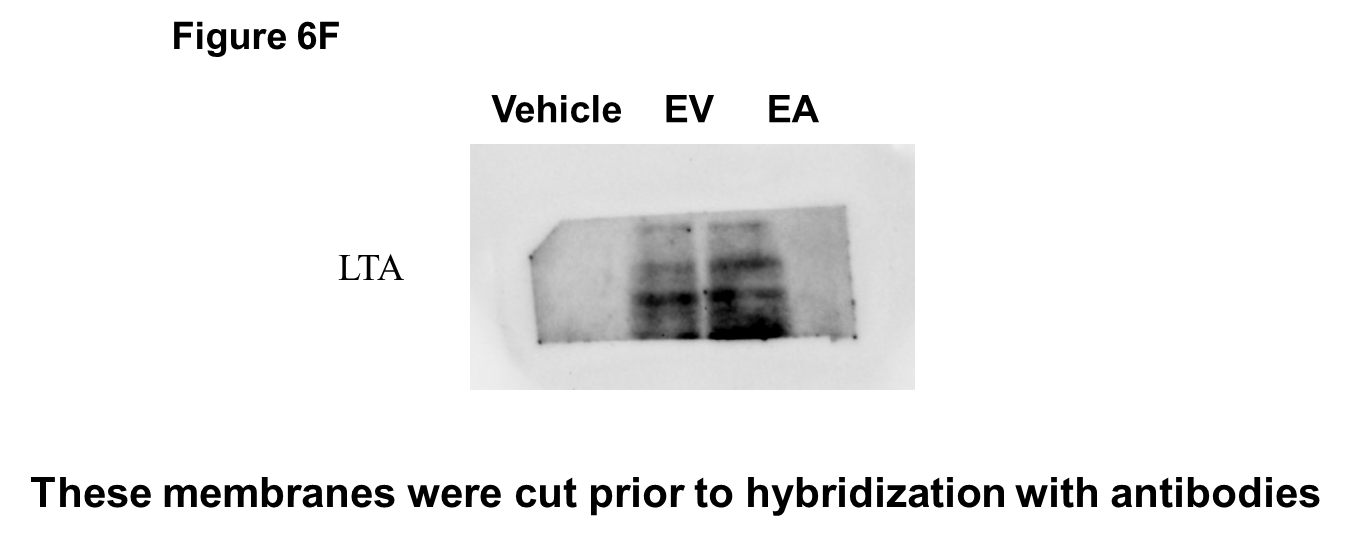


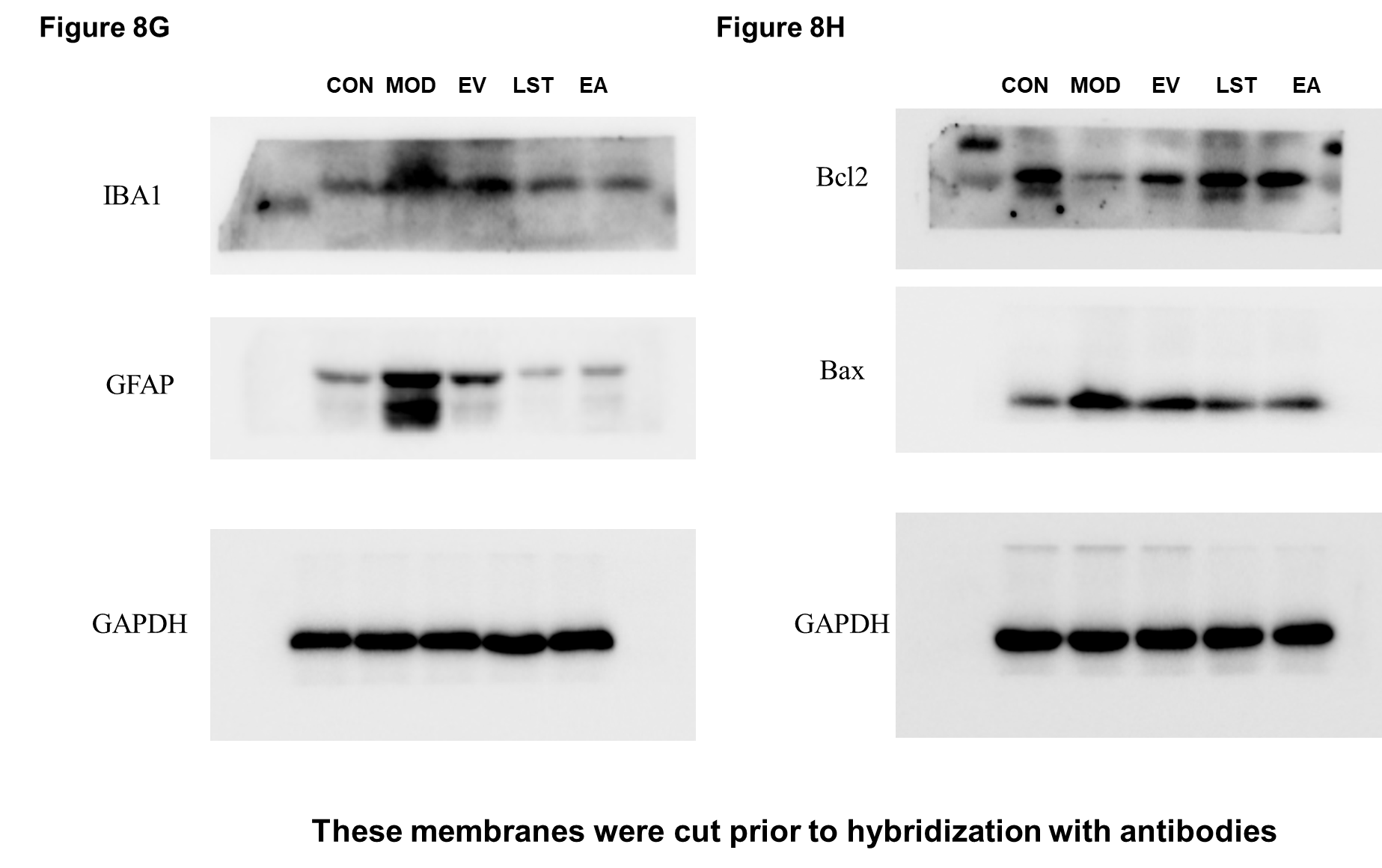


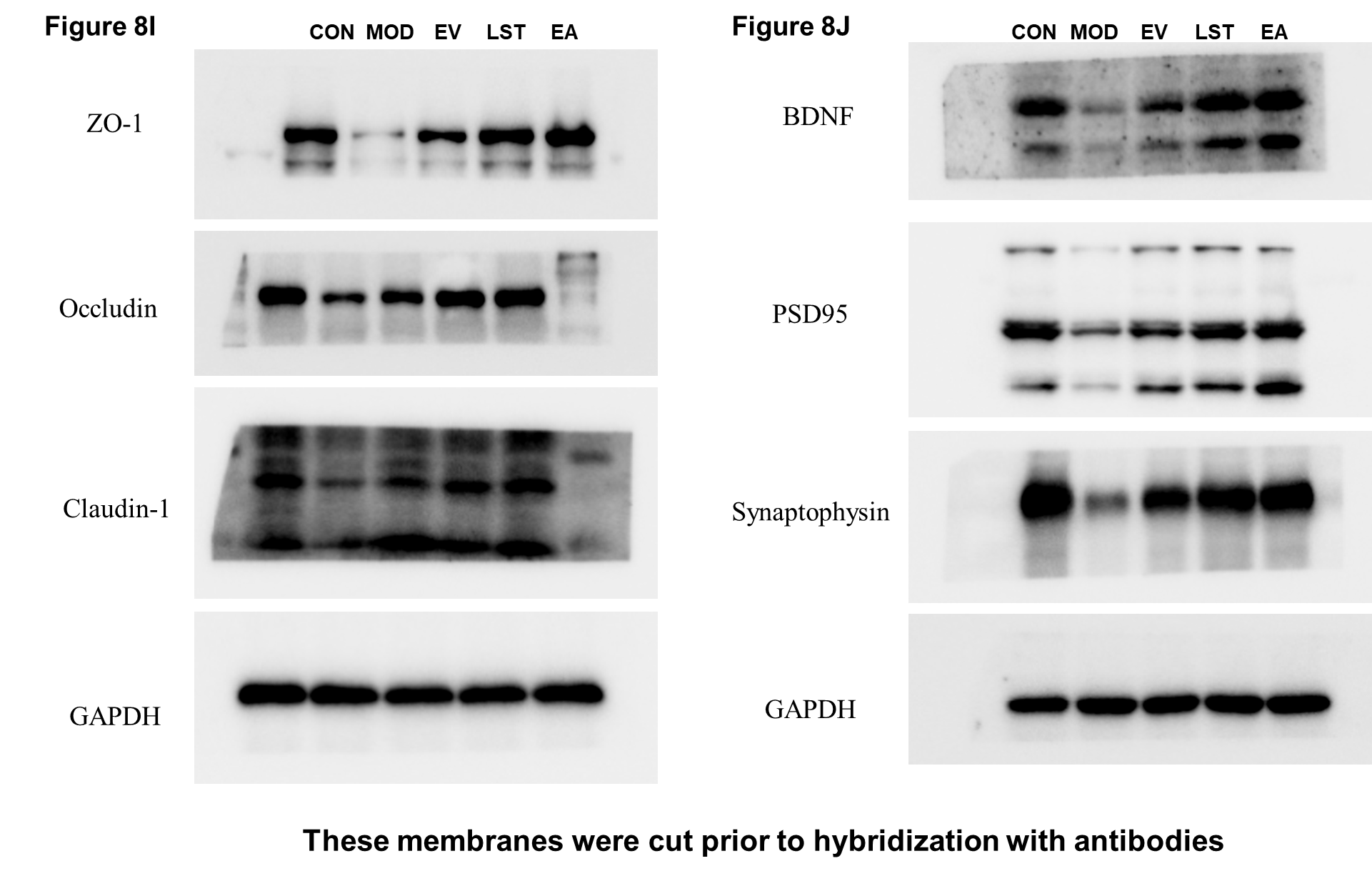


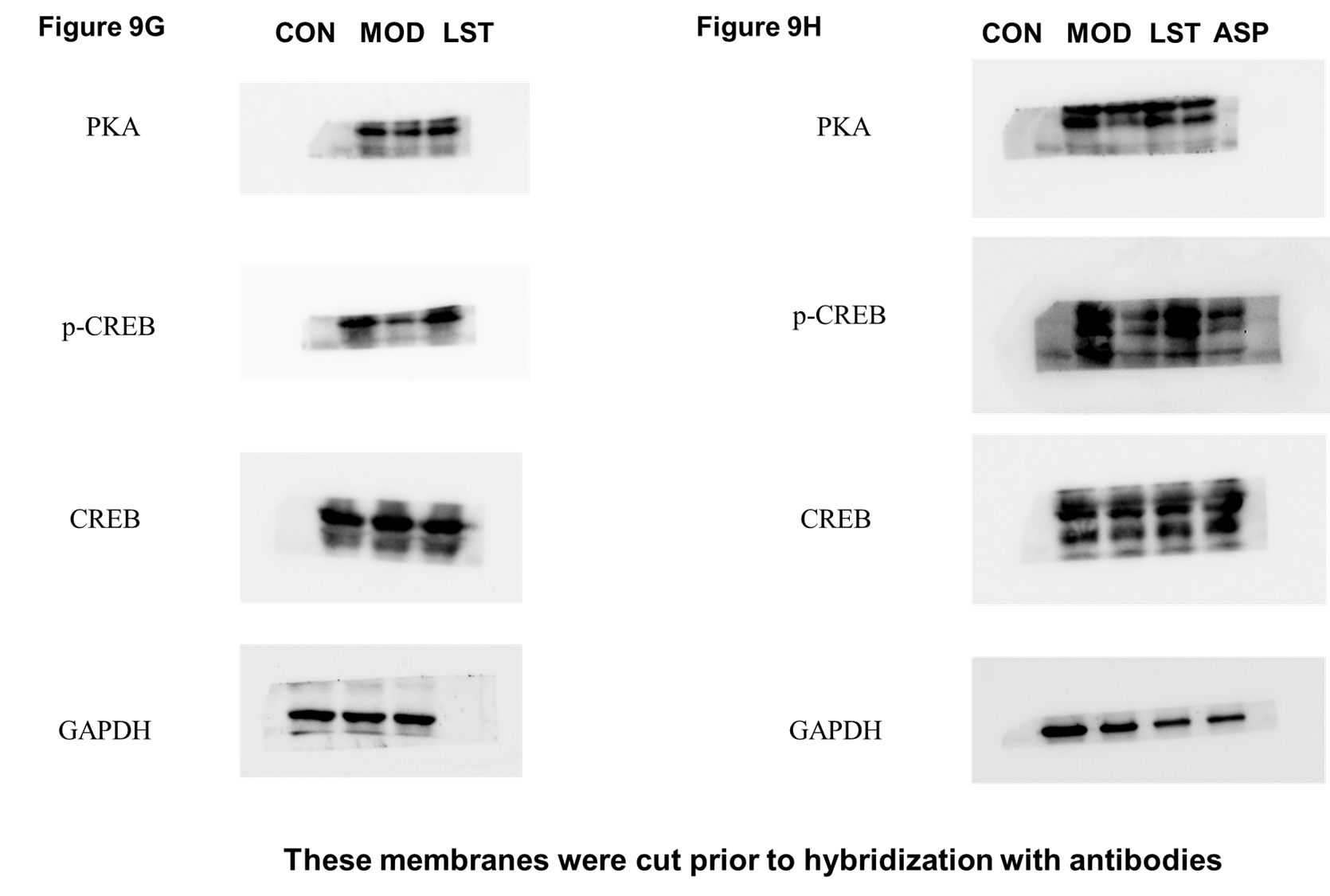


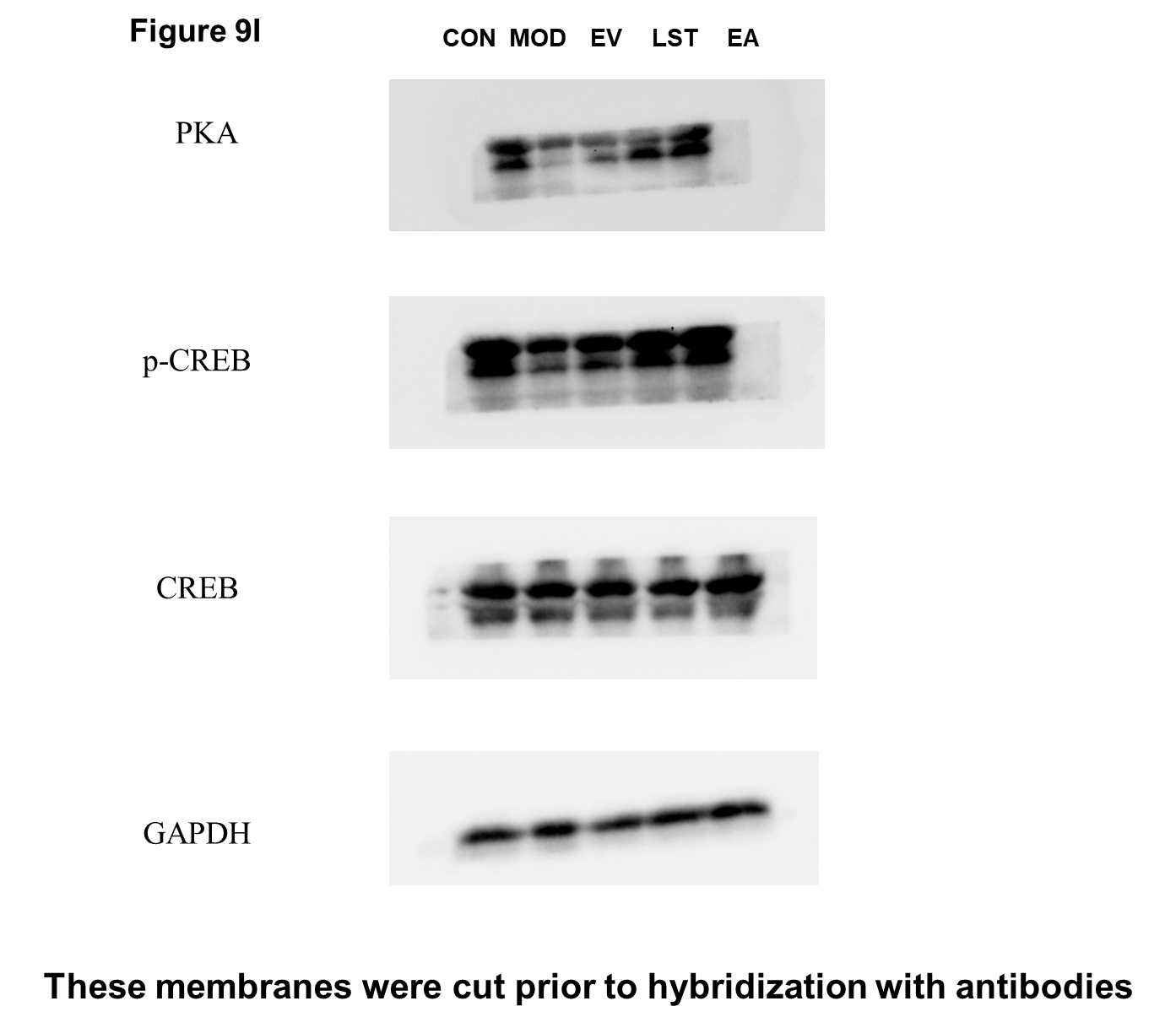


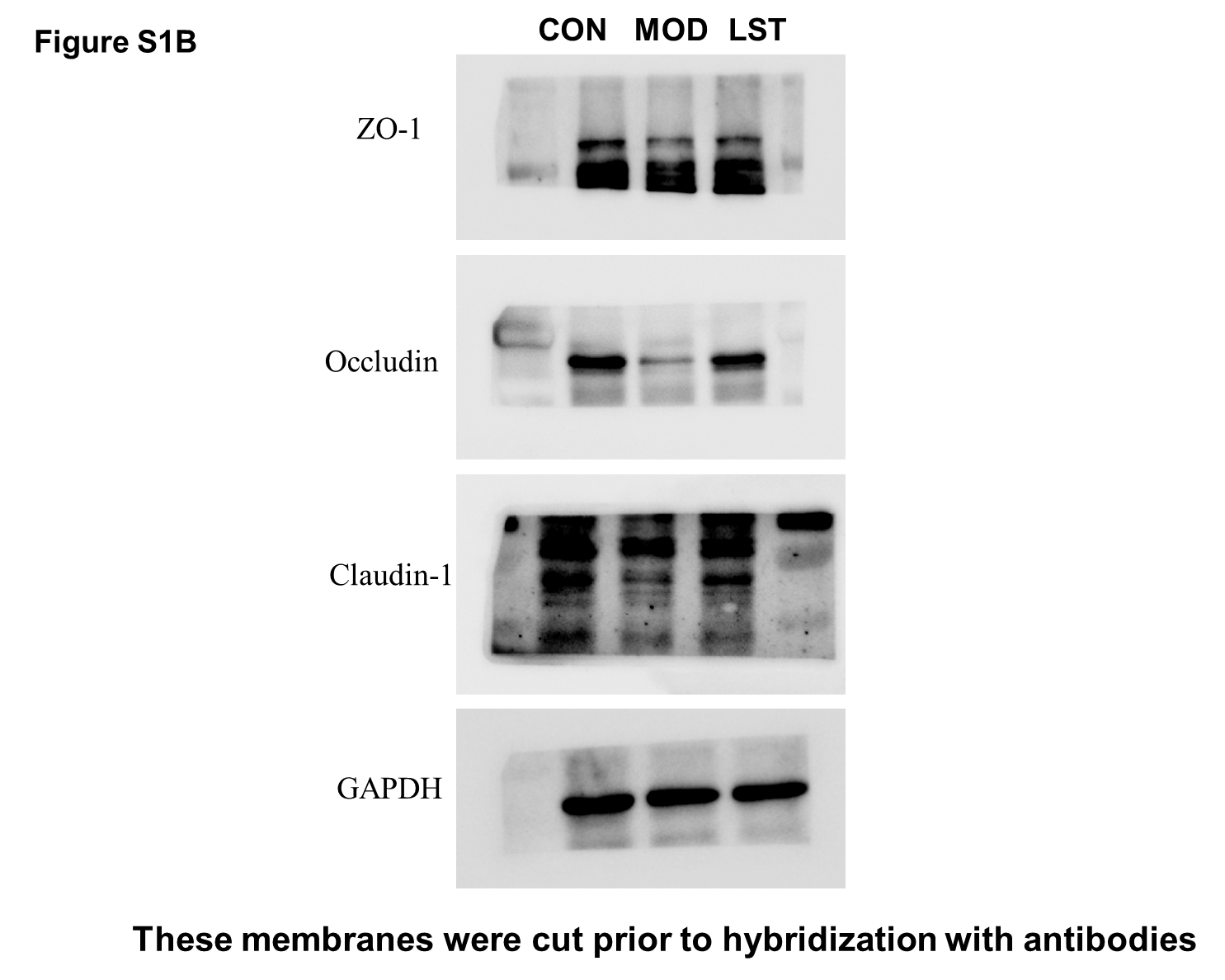


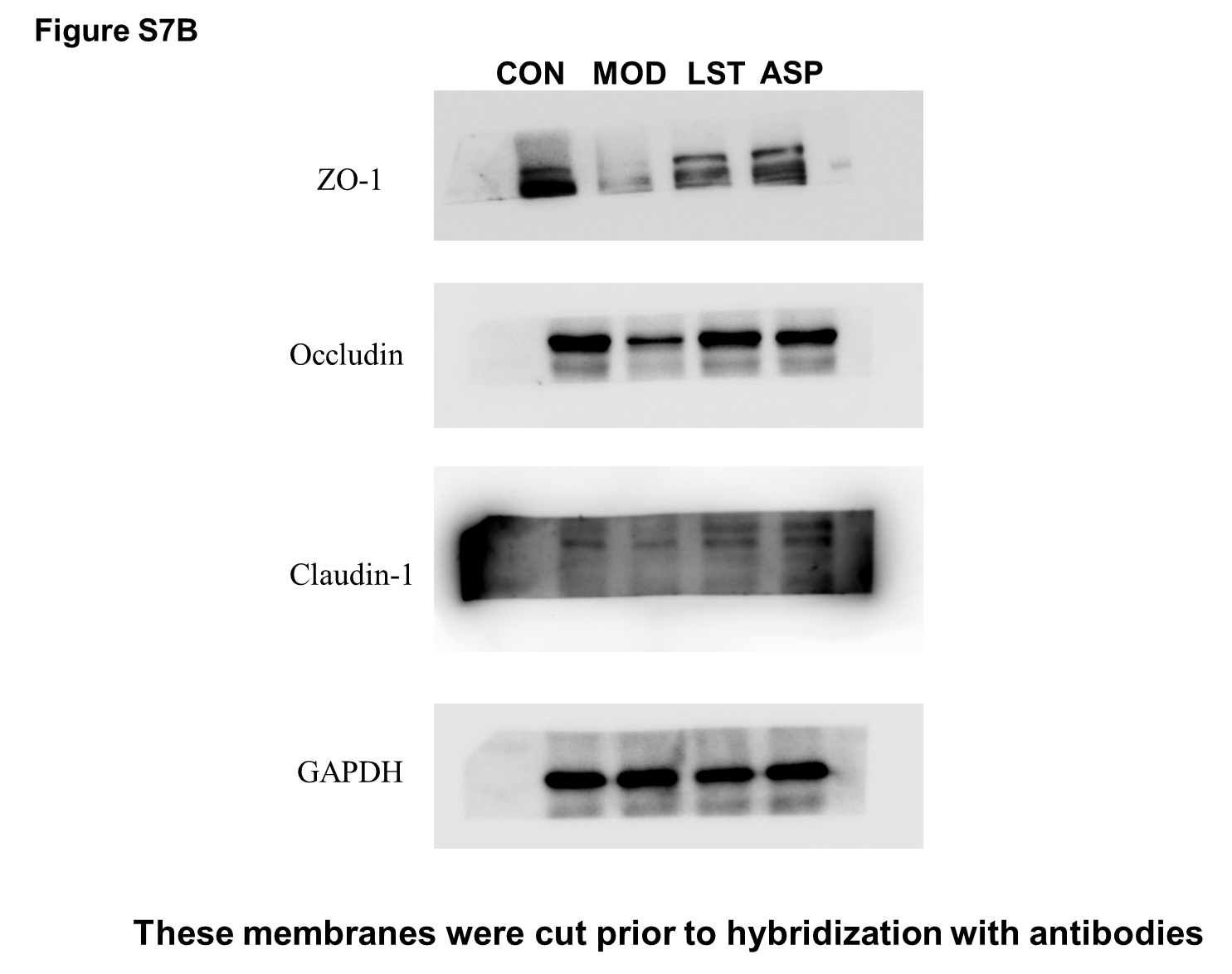


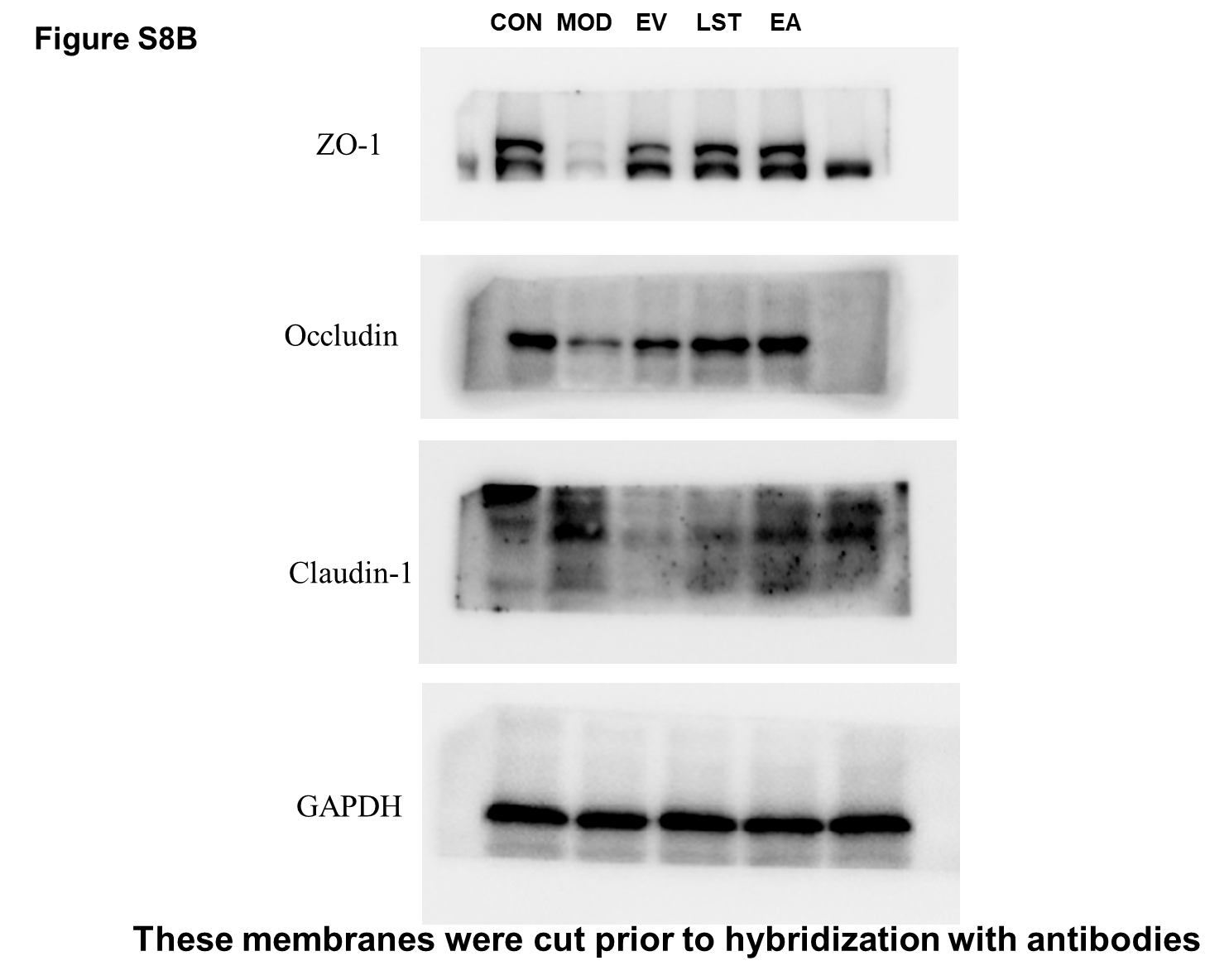

Supplement: Supplementary file 1 — Supplementary Material 1. [file 12974_2026_3864_MOESM1_ESM.docx]
