# Supplementary material for: The lung-brain axis mediates the neuroprotective effects of nasally administered L. salivarius and its EV-delivered metabolite in vascular dementia
Source: J Neuroinflammation. 2026 May 12;23:235. doi: 10.1186/s12974-026-03864-6 (PMC13343794; doi:10.1186/s12974-026-03864-6)
Supplement: Supplementary file 2 — Supplementary Material 2. [file 12974_2026_3864_MOESM2_ESM.docx]

**The Lung-Brain Axis Mediates the Neuroprotective Effects of Nasally Administered *L. salivarius* and its EV-Delivered Metabolite in Vascular Dementia**

Cihua Zheng^a, b#^, Zhuoya Wang^a, b#^, Furui Tang^a, b#^, Yuchun Zhong^b^, Jiacheng Zheng^a, b^, Jian Xie^b, e^, Li Liu^b, c^, Yimin Pi^a, b^, Xifeng Wang^d^, Tian Liu^a, b^, Zhidong He^b^, Jun Luo^a, b*^

**Affiliations**

^a^ Department of Rehabilitation Medicine, The Second Affiliated Hospital, Jiangxi Medical College, Nanchang University, Nanchang, Jiangxi, 330006, P. R. China.

^b^ Jiangxi Province Key Laboratory of Precision Cell Therapy, The Institute of Translational Medicine, The Second Affiliated Hospital of Nanchang University, Nanchang, Jiangxi, 330006, P. R. China.

^c^ Department of Traditional Chinese Medicine, The Second Affiliated Hospital, Jiangxi Medical College, Nanchang University, Nanchang, Jiangxi, 330006, P. R. China.

^d^ Department of Anesthesiology, the First Affiliated Hospital, Jiangxi medical College, Nanchang University, Nanchang, Jiangxi, 330006, P. R. China.

^e^ Department of Orthopedics, Longyan First Hospital, Longyan, Fujian 364000, PR China.

^#^These authors contributed equally to this article.

*** Correspondence:**

Jun Luo:

Department of Rehabilitation Medicine, The Second Affiliated Hospital of Nanchang University, Nanchang, Jiangxi, 330006, P. R. China, E-mail: [luojun1786@163.com](mailto:luojun1786@163.com). +8618970025500

**Materials and Methods:**

**1. Animal experiment**

SPF Sprague-Dawley (SD) rats, aged 6-8 weeks and weighing 180-220g, were procured from Tianqin Biotechnology Co., Ltd., located in Changsha City, Hunan Province. The rats were acclimated for one week at 22°C under a 12-hour light-dark cycle, with ad libitum access to food and water.

VaD rat model induced by chronic cerebral ischemia was established by ligating bilateral common carotid artery (2-VO). The procedure involved the anesthesia of rats with ether, followed by skin preparation and disinfection in the anterior cervical region. A 5 mm skin incision was made on the right side of the cervical median line to expose the right common carotid artery. Care was taken to avoid traction on the vagus nerve during blunt separation and exposure of the artery. The skin was sutured after ligation, and warm resuscitation was performed. After 48 hours, the left common carotid artery was ligated using the same method [1]. Twenty-four rats were randomly allocated into three groups (n=8/group): the control group (CON) received normal feeding; the model group (MOD) underwent nasal instillation of 100 μL physiological saline every other day. Each animal received 100 μL of formulation per nostril (total 200 μL per animal), administered via slow, alternating instillation, once every other day, for a total of 15 doses over a 30-day treatment period. the *L. salivarius* treatment group (LST) received nasal instillation of 100 μL of 1×10^8^ CFU/mL *L. salivarius* suspension every other day. To investigate the impact of ASP on VaD, an ASP treatment group was included: ASP (2 mg/kg) suspension was administered nasally every other day after model establishment. Thirty-two rats were randomly divided into four groups (n=8/group): the CON, MOD, LST and ASP group. To enhance ASP delivery efficiency, EVs derived from *L. salivarius* were utilized to encapsulate ASP, namely EVs-ASP, and the intervention effect was assessed. Twenty rats were randomly divided into five groups (n=4/group): the CON group received normal feeding; the MOD group underwent nasal instillation of 100 μL saline every other day; the LST group received *L. salivarius*; the EVs group (EV) received nasal instillation of EVs (100 μg) every other day; the EVs-ASP group (EA) received nasal instillation of EVs loaded with ASP (100 μg EVs, ASP approximately 2 mg/kg; 25% drug loading rate, ASP concentration approximately 0.4762 mg.) every other day. Following 30 days of continuous intervention, cognitive function was evaluated through behavioral tests, and subsequent sample collection was conducted for further analysis.

**2. Bacterial culture**

According to the product instructions, accurately weigh 6.62g of MRS culture medium, dissolve it in 100 mL of ultrapure water, and sterilize it under high pressure at 120℃ for 15min. Take out the frozen *L. salivarius* strain (BNCC138618, BeNa, Henan, China) from the -80℃ refrigerator, quickly recover it, and inoculate it into MRS culture medium in the super clean bench. Tighten the tube cap, gently invert and mix well, and incubate in a 37℃ shaker for 24 hours for subsequent experiments [2].

**3. Extraction and identification of bacterial EVs**

Cultivate and count *L. salivarius*, take the bacterial solution and centrifuge twice (30min each time) at 4°C and 5000g to obtain the supernatant. The supernatant is filtered through 0.45 μm and 0.22 μm filters respectively, and then concentrated using a 100 kDa Amicon Ultra-15 filter by centrifugation at 5000 ×g for 30 min at 4 ℃. The concentrated solution is centrifuged at 4°C and 150000×g for 90 min, and the supernatant is discarded to obtain EVs containing a small number of impurities; When purifying, wash the precipitate three times with sterile PBS and resuspend it well. Mix it with OptiPrep solution (60% v/v) to make a 55% (v/v) mixture. Transfer it to an ultracentrifuge tube and add OptiPrep solution at high to low concentrations.

Centrifuge at 4°C and 100000×g for 18 h. Take 1 mL of each layer and dilute it with NTA to determine the density layer rich in vesicles. Take the liquid from this layer and centrifuge at 4°C and 150000×g for 2 hours. Wash the precipitate three times with sterile PBS to obtain purified EVs and pack them separately. NTA detection requires diluting purified EVs with PBS to 30-100 particles per frame, passing them through a 532 nm laser via an injection pump, and analyzing and recording their particle size distribution and quantity using NTA software. The following are the specific parameters of NTA test: Imaging & capture settings: 3 independent captures were performed, with a camera exposure of 31.00 ms, contrast gain of 5.35, focus capture position of 4805, frame rate of 32.2091 fps, and a capture duration of 23.3 seconds (750 frames) per capture. Instrument & algorithm details: Measurements were conducted on a Malvern Panalytical NS XPLORER instrument (serial number: 600000045) using a 488 nm laser (light scatter filter), flow rate of 0.0 μL/min, and analyzed via the FTLA distribution algorithm (software version 1.1.0.6). These parameters ensure the reproducibility and reliability of our exosome characterization results. Subsequently, EVs were dropped onto a copper mesh with a supporting membrane, allowed to stand at room temperature for 1 min, and excess liquid was removed using filter paper. When the sample was almost dry, uranyl oxalate solution was added dropwise for negative staining. After standing at room temperature and drying, EVs images were observed and obtained by TEM [3].

**4. EA drug loading**

1 mg ASP and diluted EV were mixed ultrasonically in ice bath, and the parameters were set to 500 V, 2 kHz and 20% power. Six cycles were treated in pulse mode of 2 s on /2 s off, and cooled on ice for 2 min between cycles. After ultrasound, the samples were incubated at 37°C for 2–3 hours to recover the structure, and then centrifuged at 12,000 g for 10 min to remove the unloaded drugs. The content of ASP in EA was determined by ultraviolet spectrophotometry at 238 nm, and the standard curve was established with 0.0078-0.125 mg/mL ASP standard solution for calculation. The encapsulation efficiency is calculated according to the formula EE = We/Wt × 100%, and the drug loading is calculated according to LE = We/(Wt+Ws) × 100%, where Wt is the total amount of ASP, We is the quality of ASP loaded, and Ws is the quality of EV protein estimated by BCA method.

**5. Zeta potential analysis**

Collect appropriate samples (EV and EA) and dilute them to 0.01%-0.1% using suitable solvents. Ensure thorough mixing by vortexing or subjecting to 300-500W ultrasound for 1-3 min. Preheat the Zeta potentiometer for 30 min and calibrate it with a standard solution. Wash the sample cell meticulously with the samples, inject the samples carefully to prevent bubble formation, and place them in the instrument. Set parameters such as temperature (e.g., 25℃, adjustable as necessary), initiate the detection process, and automatically record data 3-5 times to obtain an average value .

**6. Organizational distribution**

The detection flow of tissue distribution in EVs and EAs is as follows: According to the above method, EVs and EAs were labeled with Dil and PKH26 respectively and resuspended with PBS. Then SD rats fasted for 24h were given nasal administration. The femur, tibia, kidney, liver, lung, heart, spleen and brain were collected in Dil labeled group at 3, 12 and 48h after administration, and photos were taken with IVIS Lumina (Tanon ABL X6, Shanghai, China). In PKH-26 labeled group, frozen sections were taken from tissues 3h after administration, and the distribution of EVs and EAs in tissues was observed by fluorescence microscope after DAPI staining [3].

**7. Behavioral experiments**

**7.1. Three-Chamber Social Test**

The Three-Chamber social experiment device is a 90×40×30cm cuboid box, which is divided into three equal-sized rooms (left, middle and right) by plexiglass. A hollow metal cage is placed in the left and right rooms for placing strange rats. The experiment was carried out in a quiet environment, and the whole process was recorded, and the device was disinfected between each link to avoid odor interference. The experiment is divided into three stages: adaptation stage, put the rats in the central room facing back, open the partition and let them explore the three rooms freely for 10 min. social test, put a strange rat (S1) into one cage, and the other cage is empty (E). Put the rats in the central room again, move freely for 10 min, and record their social behavior with strange rats. Social novelty test, put a second strange rat (S2) in the other cage, and keep S1 in the original cage. Similarly, the mice were placed in the central room, and their interactions with two strange mice were recorded within 10 min to evaluate their social preference and social novelty preference test. Social Preference Index=(S1-E)/(S1+E); Social Novelty Preference Index=(S2–S1)/(S2+S1) [4].

**7.2. Open field test**

Open field test is a classic experimental method to evaluate the anxiety and depression of animals, which is used to explore the changes of cognitive ability and emotional state of animals in nervous system disease models. The open field experiment box is a square open box with 100×100×50cm. It is equipped with a video tracking system, which is used to record the movement track of rats. In the computer software, it is set as 25 square squares with equal size, with 16 squares on the periphery and 9 squares in the center. Firstly, a dark and quiet atmosphere should be created. Rats should be put into an open-field experimental box to adapt to the environment for 5 min, and their movement tracks should be recorded within 5 min after adaptation. Following the experiment, it is imperative to disinfect each rat using 75% alcohol to eliminate any odor that could potentially interfere with subsequent experiments. The system software was utilized to record parameters such as the total distance of autonomous movement, as well as the time and distance of movement within the central area by the rats [5].

**7.3 Morris water maze experiment**

The Morris water maze experiment assesses animals' spatial learning and memory capabilities using a circular pool, a hidden platform, and a video tracking system. The pool is partitioned into four quadrants, with the platform positioned centrally in one quadrant, submerged 2cm below the water's surface. The water temperature is maintained at approximately 25°C, with consistent ambient lighting. The experiment comprises two phases. During the initial phase spanning five days, rats undergo training in spatial navigation. Each day, rats are placed in the water from different quadrant entry points, and their time to locate the hidden platform (escape latency) and movement trajectory are recorded. Rats failing to find the platform within 120s are guided to it and allowed to remain for 30s. The subsequent phase, conducted on the sixth day, involves a probe trial to assess spatial memory retention. Following platform removal, rats are released into the water from the quadrant opposite the original platform and permitted to swim freely for 120s. Their movement paths, frequency of crossing the initial platform location, and escape latency are recorded to evaluate spatial memory retention [1].

**7.4. Elevated plus maze test**

The experimental device of the elevated plus maze is mainly composed of two open arms (45×15cm), two closed arms (45×15×30cm) and a central area (15×15cm). The device is 70cm away from the ground, and a monitoring camera is equipped above the device to capture the activity data of rats, which are analyzed by the connected animal behavior analysis software. After adapting to the environment, the rats were taken out of the cage, put into the central area with their heads facing the open arms, and recorded their activities within 5 min. The frequency and duration of entries into the open and closed arms were analyzed [6].

**7.5. Sucrose preference experiment**

The sucrose preference experiment was conducted to determine the preference of rats to sucrose solution under the condition of free choice through four days of adaptation, fasting and testing. The specific process includes: adapting to sucrose solution and ordinary water for two days, fasting on the third day, and drinking test for one hour on the fourth day. Sucrose preference ratio: sucrose preference ratio= (sucrose intake/total intake) ×100%, where the total intake is the sum of sucrose solution intake and water intake [5].

**8. Quantitative Polymerase Chain Reaction (qPCR)**

Take rat brain tissue homogenate, extract total RNA with TRIzol reagent (Invitrogen), then reverse transcribe RNA into cDNA with all-in-one first strand cDNA synthesis kit II (with dsDNA SE). The reaction system and procedure refer to the kit instructions, and the product is diluted with ribozyme-free water for later use. Next, according to the instructions of PCR kit, amplification was carried out in PCR instrument by three-step method. The primer sequences are shown in the following table. The reaction system and procedure refer to the above steps, and the gene expression was normalized by 2-ΔΔCT method with GAPDH as internal reference [6].

The primer sequences required for rat target genes and internal reference genes are shown below.

| Name | primer sequence |
| --- | --- |
| TNF-α-Forward Primer | CCACCACGCTCTTCTGTCTACTG |
| TNF-α-Reverse Primer | TGGGCTACGGGCTTGTCACT |
| IL-6-Forward Primer | AGGATACCACCCACAACAGACC |
| IL-6-Reverse Primer | TTGCCATTGCACAACTCTTTTC |
| IL-1β-Forward Primer | TGACCTGTTCTTTGAGGCTGAC |
| IL-1β-Reverse Primer | CATCATCCCACGAGTCACAGAG |
| GAPDH-Forward Primer | CTGGAGAAACCTGCCAAGTATG |
| GAPDH-Reverse Primer | GGTGGAAGAATGGGAGTTGCT |

**9. Western blot**

The protein extraction of brain tissue and lung tissue adopts the cracking system of RIPA lysate (Cat# R0010, Solarbio) and PMSF (Cat# P0100, Solarbio) in a ratio of 100:1. The extracted protein samples were quantified by BCA protein concentration detection kit (Cat# PC0020, Solarbio). Subsequently, the protein samples were separated by 6%–15% polyacrylamide gel electrophoresis and transferred to PVDF membrane at a constant current of 260 mA. After the membrane transfer, the PVDF membrane was sealed with 5% skimmed milk (Cat#P1622-1, Beijing Applygen Technologies) on a room temperature shaker for 90 minutes. After that, the membrane was incubated with the following antibodies at 4℃ overnight: rabbit anti-GFAP (Cat#16825-1-AP); rabbit rabbit anti-IBA1 (Cat# 21773-1-AP); mouse anti-Bax (Cat# 50599-2-Ig); rabbit anti-Bcl-2 (Cat#68103-1-Ig); rabbit anti-BDNF (Cat#25699-1-AP); rabbit anti-PSD95 (Cat#20665-1-AP); rabbit anti-SYP (1:4000; Cat#17785-1-AP); rabbit anti-ZO-1 (Cat#21773-1-AP), rabbit anti-Occludin (Cat#27260-1-AP); rabbit anti-Claudin-1 (Cat#131050-1-AP) and mouse anti-GAPDH (Cat#60004-1-Ig). After washing the membrane with TBST for three times, they were incubated with goat anti-rabbit secondary antibody (1:5000, Cat# bs-0295G-HRP, Bioss) or goat anti-mouse secondary antibody (Cat#bs-0296G-HRP, Bioss) labeled with HRP for 1h at room temperature. Finally, the high sensitivity enhanced chemiluminescence reagent (Cat# PA112, Tiangen Biotechnology) was used for development detection [7].

**10.Tissue Staining**

Brain and lung tissues from rats were harvested and fixed in 4% paraformaldehyde for 48 hours. The tissues were dehydrated using a gradient of alcohol, made transparent with xylene, and embedded in paraffin blocks after gradual waxing at 60℃. Subsequently, 4μm sections were cut and dried at 37℃. The sections were dewaxed in xylene, hydrated with a gradient of alcohol, and stained with hematoxylin for 5 min. Differentiation with hydrochloric acid in alcohol and turning blue with lithium carbonate followed. Eosin staining for 5 minutes, dehydration, transparency, sealing, and staining with Hematoxylin and Eosin (HE) were performed. Images were captured using a 200× microscope. For Nissl staining, sections were dewaxed, hydrated, and immersed in 1% toluidine blue at 37℃ for 15 min. Differentiation with 95% alcohol, dehydration, and sealing with Nissl stain were carried out [7]. The sections were observed and photographed under a microscope. After dewaxing and hydration, antigen retrieval was performed using citrate buffer. The sections were sealed with 5% fetal bovine serum for 1 hour. Brain tissue sections were treated with BDNF (1:100), PSD-95 (1:100), SYP (1:200), NeuN (1:200), GFAP (1:200), and IBA1. Lung tissue sections were incubated with ZO-1 (1:100), Occludin (1:200), and Claudin-1 (1:200). Subsequently, they were treated with a fluorescent secondary antibody (1:100) in the dark for 1h, sealed with DAPI, stained with immunofluorescence double labeling, and imaged using a fluorescence microscope [8].

**11. Golgi staining**

Rat brain tissue was fixed in Golgi staining fixative (Servicebio G1069) and stored at room temperature. The fixative was then replaced with Gorky dye solution, and the tissue was stained at 26°C for 14 days. The dye solution was changed 48h after initiation of staining and subsequently every 3 days. Following staining, the brain tissue underwent a 1-hour treatment in tissue treatment solution, followed by a 3-day treatment at 4°C in darkness with fresh solution. The tissue was then sectioned into 60 μm slices using a vibrating microtome and mounted on glass slides. The slices were rinsed with ultrapure water, treated with developer for 30 min, rinsed again, sealed with glycerol gelatin, and stored in darkness. Golgi morphology was observed using an optical microscope, and images were captured [9].

**12. ELISA**

Peripheral serum of rats was collected and centrifuged at 4°C and 1000×g for 15 min. Then, according to the kit instructions, the concentrations of IL-1β, TNF-α and IL-6 in serum were quantitatively detected by ELISA kits (IL-1β: Cat# RK00009; IL-6: Cat# RK00020; TNF-α: Cat#RK00029; all purchased from ABClonal, China).

**13. DNA extraction and 16S rRNA gene sequencing**

The frozen lung tissue was taken out and the total gene DNA of rat lung microorganism was extracted according to the instructions of genome DNA kit. The variable V3-V4 region of the 16S rRNA gene (338F, 5′- ACTCCTACGGGAGGCAGCA-3′; 806R, 5′- GGACTACHVGGGTWTCTAAT-3 ′) was amplified for each sample using primers 338F/806R. The PCR products were sequenced using Illumina platform and aligned with known 16S rRNA gene databases to determine the microbial species to which the sequences belonged. According to the results, the relative abundance of different microorganisms in the samples was counted to analyze the composition structure of microbial community in rat lung tissue, and the α diversity index and β-diversity index were calculated to evaluate the diversity of microbial community in rat lung tissue and the difference between samples [2].

**14. Untargeted metabolomics:**

50 mg of rat brain and lung tissue samples were weighed and homogenized in a high-throughput tissue grinder with 500 μL of extraction solution (acetonitrile: isopropanol: water=3:3:2). The homogenate was then supplemented with an equal volume of extraction solution, sonicated in an ice water bath for 5min, and centrifuged at 10,000g for 5min. The resulting supernatant was collected, concentrated to dryness under vacuum for 8-10h, reconstituted in 80 μL of a 20 mg/mL methoxypyridine solution, vortexed for 30s, and incubated at 60°C for 60min. Subsequently, 100 μL of BSTFA-TMCS (99:1) was added, and the mixture was incubated at 70°C for 90 minutes, followed by centrifugation for 5min. The supernatant was then sent to Parthenol Biotech (Shanghai) for GC-TOF-MS analysis using a DB-5MS column with a flow rate of 1 mL/min. A 1 μL sample was injected at a 1:10 split ratio with an injection port temperature of 280°C. The transmission line and ion source temperatures were set at 320°C and 230°C, respectively. The temperature program started at 50°C for 30s, followed by a ramp of 15°C/min to 320°C, which was maintained for 9min. Mass spectrometry was performed at a rate of 10 spectra/s with an electron energy of -70 V and a 3-minute solvent delay for full scan. Subsequent experiments and bioinformatics analyses were conducted upon sample qualification [3].

**15. Targeted metabolomic analysis of SCFAs**

Rat brain and lung tissues were rapidly frozen in dry ice. A gradient mixture containing seven short-chain fatty acid standards (SCFAs) was prepared to establish a standard curve based on the chromatogram. Approximately 50 mg of the quick-frozen tissue was homogenized after grinding, followed by ultrasonication in an ice-water bath for 30min. The homogenate was then centrifuged at 4℃ and 10000×g for 15min to collect the supernatant. Subsequently, ethyl acetate was added to the supernatant, vortex mixed, ultrasonicated in an ice-water bath for 10min, and centrifuged at 4℃ and 10000×g for 10min. The resulting supernatant was analyzed for the concentrations of the seven SCFAs using gas chromatography-mass spectrometry [10] .

**16. RNA-seq analysis**

Total RNA was extracted from rat brain tissues in the MOD and LST groups. Library preparation and sequencing were conducted by Personalbio Biotechnology Co., Ltd. High-throughput sequencing data were obtained and aligned against reference databases to identify transcripts. Transcript data were functionally annotated using the Gene Ontology (GO), Kyoto Encyclopedia of Genes and Genomes (KEGG), Enzyme Commission (EC), EggNOG, and UniProt databases. After data normalization, differential gene expression analysis was carried out. Transcripts with a *P*-value < 0.05 and fold change (FC) > 1.5 were considered statistically significant and used as the primary criteria for identifying differentially expressed genes [11].

**17. Statistical Analysis:**

The data analysis and processing of this study are all completed by GraphPad Prism 9.0 software. The data are expressed as mean±SD. For the comparison between groups, One-Way ANOVA was used. The significance level is set to *P* < 0.05, which means that there is a statistical difference. The following symbols are used in the result chart to indicate the significance level of the difference: **P* < 0.05, ***P* < 0.01, ****P* <0.001 [3].


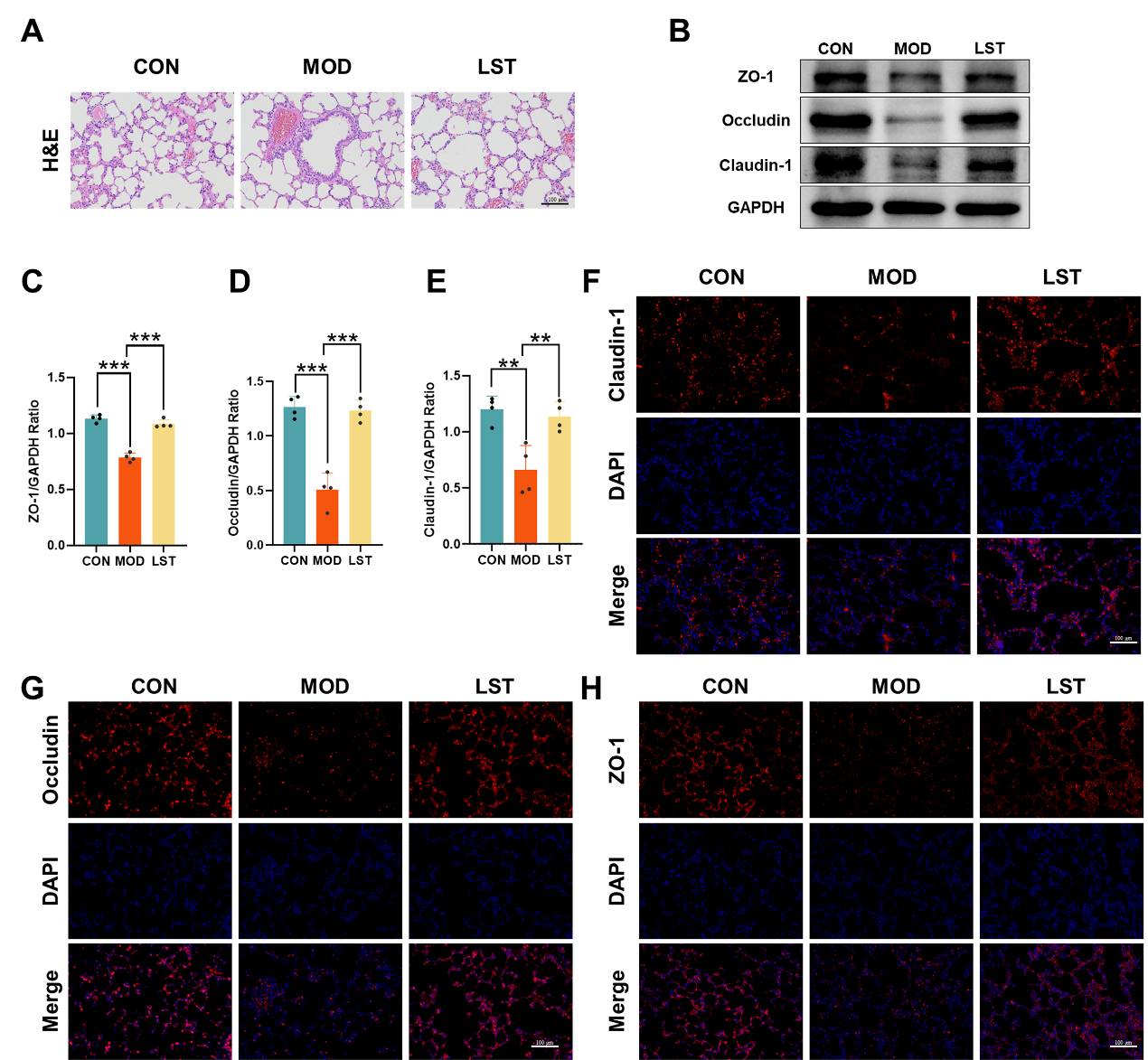


**Figure S1. Effects of *L. salivarius* on lung tissue integrity in VaD rats.** (A) H&E staining of lung tissue. Scale bar: 100 μm. (B-E) Protein expression of ZO-1, Occludin and Claudin-1 in lung tissue. (F-H) Immunofluorescence of ZO-1, Occludin and Claudin-1 in lung tissue. Scale bar: 100 μm. CON, normal control. MOD: VaD rats treated with normal saline every other day. LST: VaD rats administered *L. salivarius* every other day. Data are presented as mean ± SEM. ***P* < 0.01, ****P* < 0.001.


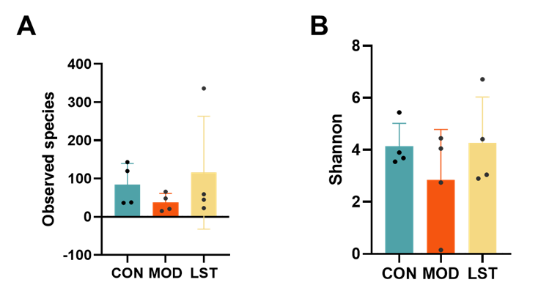


**Figure S2. α-Diversity analysis of pulmonary microbiota.** (A) Chao1 index. (B) Shannon index. CON, normal control. MOD: VaD rats treated with normal saline every other day. LST: VaD rats administered *L. salivarius* every other day. Data are presented as mean ± SEM. No significant differences were observed between groups (*P* > 0.05).


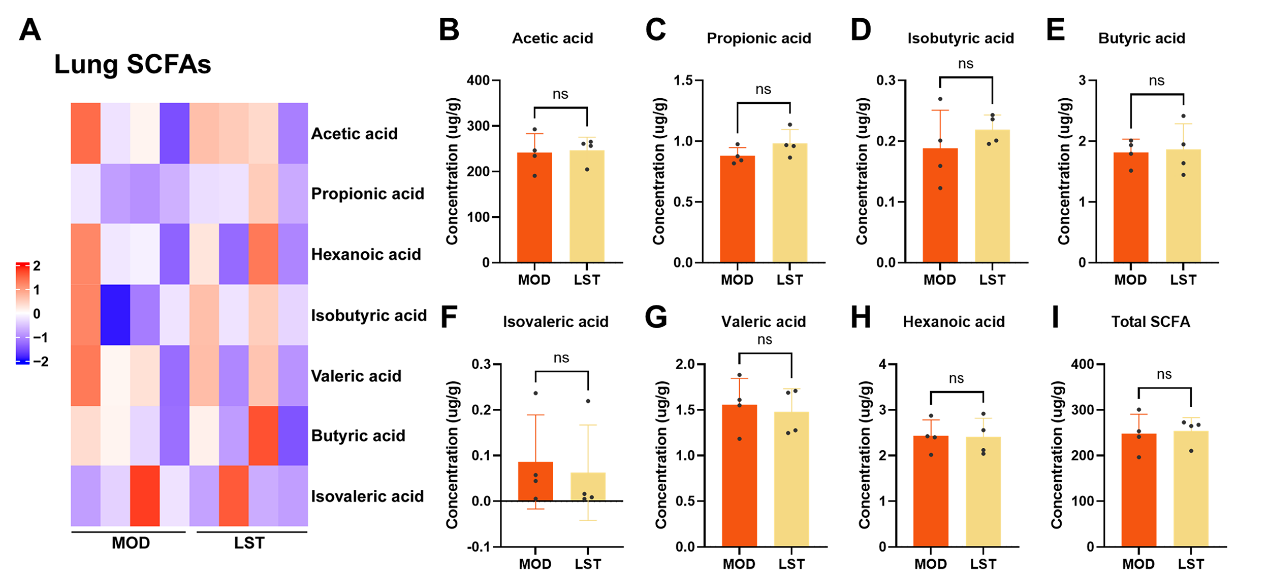


**Figure S3. Short-chain fatty acids (SCFAs) levels in lung tissue.** (A) Heat map of SCFAs. (B) Acetic acid. (C) Propionic acid. (D) Isobutyric acid. (E) Butyric acid. (F) Isovaleric acid. (G) Valeric acid. (H) Hexanoic acid. (J) Total SCFA. MOD: VaD rats treated with normal saline every other day. LST: VaD rats administered *L. salivarius* every other day. No significant differences were detected between groups (*P* > 0.05).


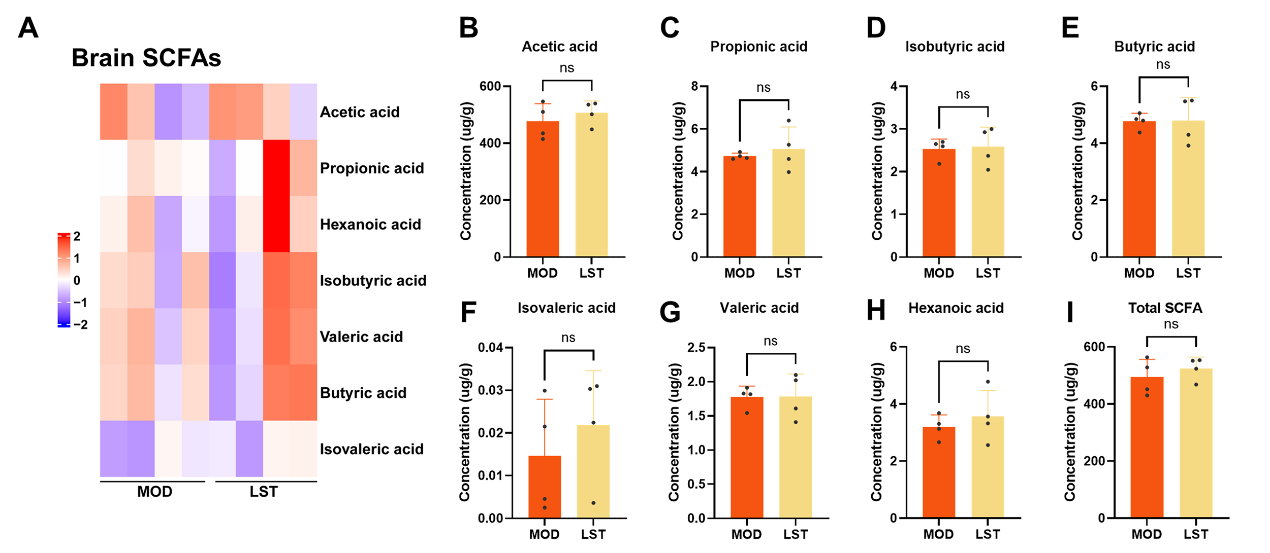


**Figure S4. SCFAs levels in Brain tissue.** (A) Heat map of SCFAs. (B) Acetic acid. (C) Propionic acid. (D) Isobutyric acid. (E) Butyric acid. (F) Isovaleric acid. (G) Valeric acid. (H) Hexanoic acid. (J) Total SCFA. MOD: VaD rats treated with normal saline every other day. LST: VaD rats administered *L. salivarius* every other day. No significant differences were detected between groups (*P* > 0.05).


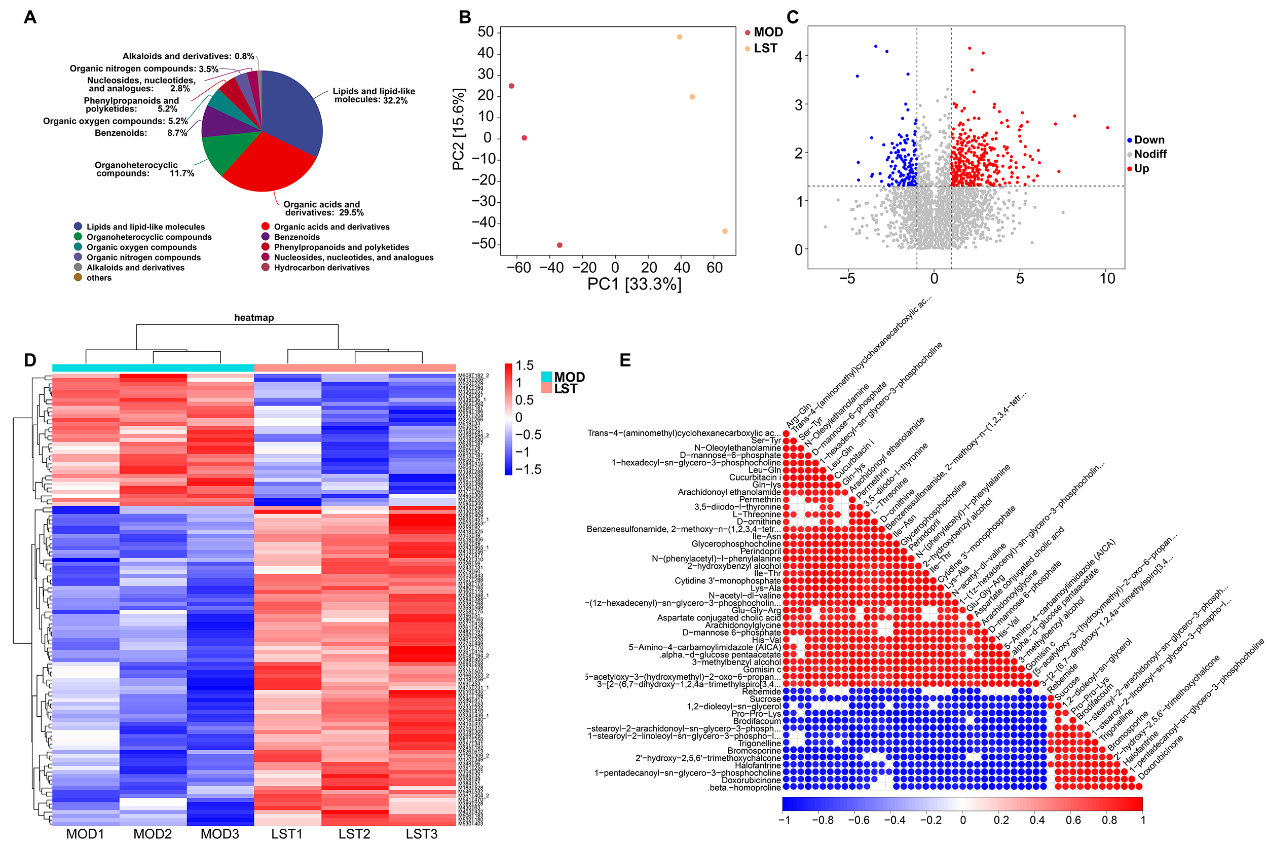


**Figure S5. Lung tissue metabolomic profiling.** (A) Composition of metabolite classes. (B) PCA score plot showing separation between MOD and LST groups. (C, D) Volcano plot and Heatmap of differentially abundant metabolites (VIP > 1, *P* < 0.05).

(E) Heatmap of differential metabolite associations. MOD: VaD rats treated with normal saline every other day. LST: VaD rats administered *L. salivarius* every other day.


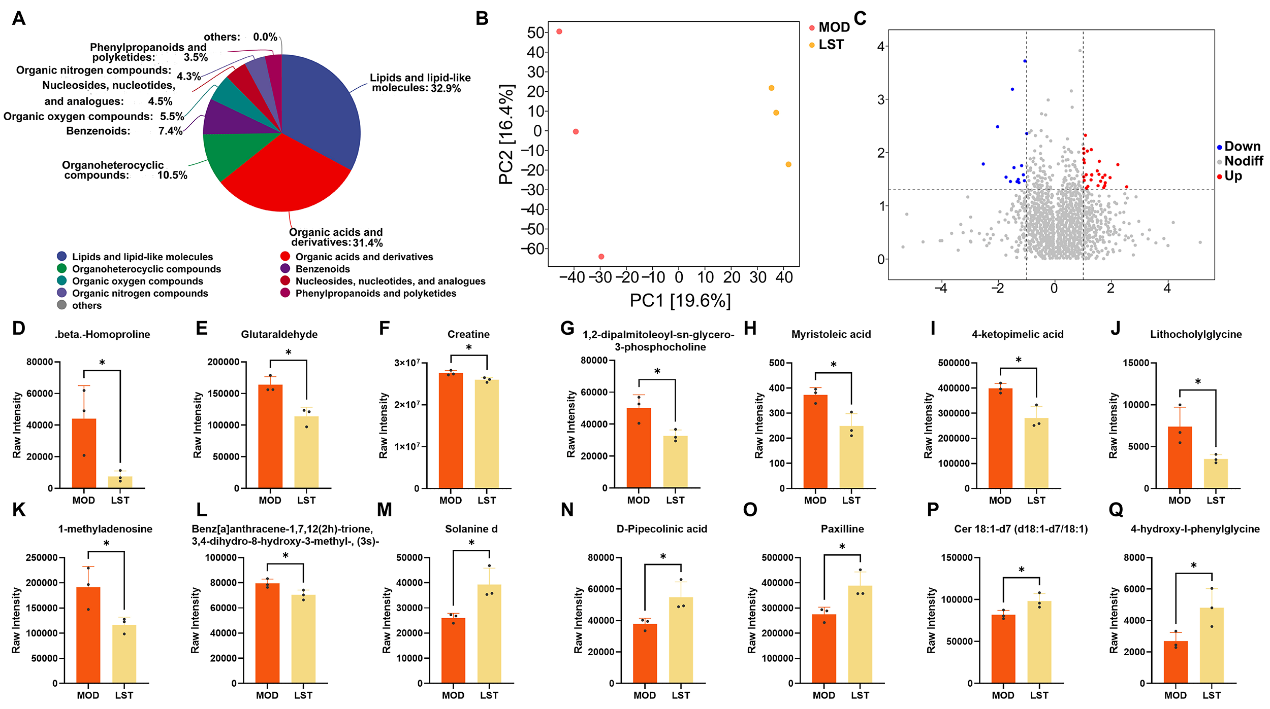


**Figure S6. Brain tissue metabolomic profiling.** (A) Composition of metabolite classes. (B) PCA score plot. (C) Volcano plot of differentially abundant metabolites. Raw intensity of (D) beta -Homoproline. (E) Glutaraldehyde. (F) Creatine. (G) 1,2-dipalmitoleoyl-sn-glycero-3-phosphocholin... (H) Myristoleic acid. (I) 4-ketopimelic acid. (J) Lithocholylglycine. (K) 1-methyladenosine. (L) Benz[12]anthracene-1,7,12(2h)-trione, 3,4-dihy... (M) Solanine d. (N) D-Pipecolinic acid. (O) Paxilline. (P) Cer 18:1-d7 (d18:1-d7/18:1) (Q) 4-hydroxy-l-phenylglycine. MOD: VaD rats treated with normal saline every other day. LST: VaD rats administered *L. salivarius* every other day. Data are presented as mean ± SEM. **P* < 0.05.


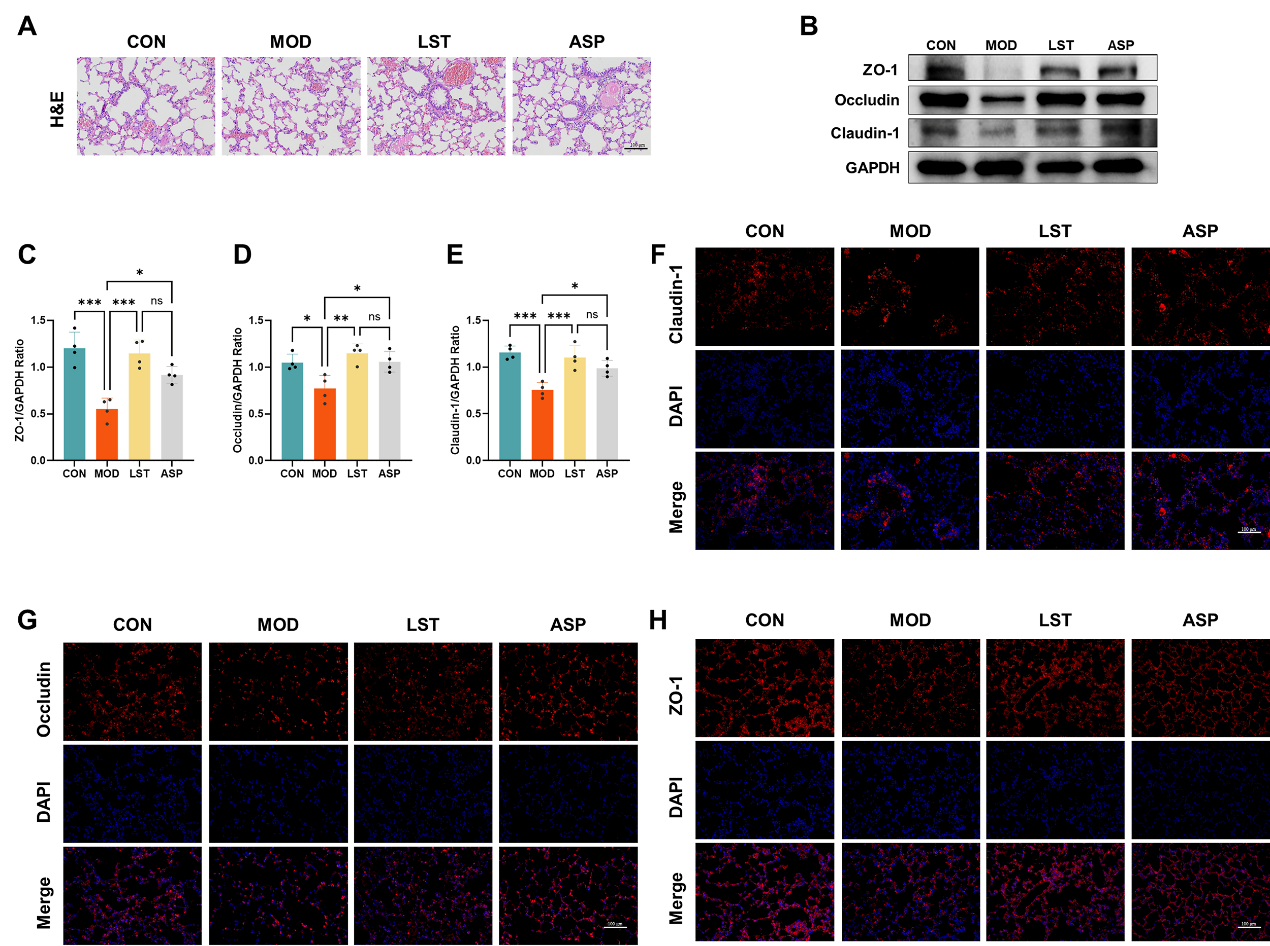


**Figure S7. Effects of ASP on lung tissue integrity in VaD rats.** (A) H&E staining of lung tissue. Scale bar: 100 μm. (B-E) Protein expression of ZO-1, Occludin and Claudin-1 in lung tissue. (F-H) Immunofluorescence of ZO-1, Occludin and Claudin-1 in lung tissue. Scale bar: 100 μm. CON, normal control. MOD: VaD rats treated with normal saline every other day. LST: VaD rats administered *L. salivarius* every other day. ASP: VaD rats administered ASP every other day. Data are presented as mean ± SEM. ns. *P* > 0.05, **P* < 0.05, ***P* < 0.01, ****P* < 0.001.


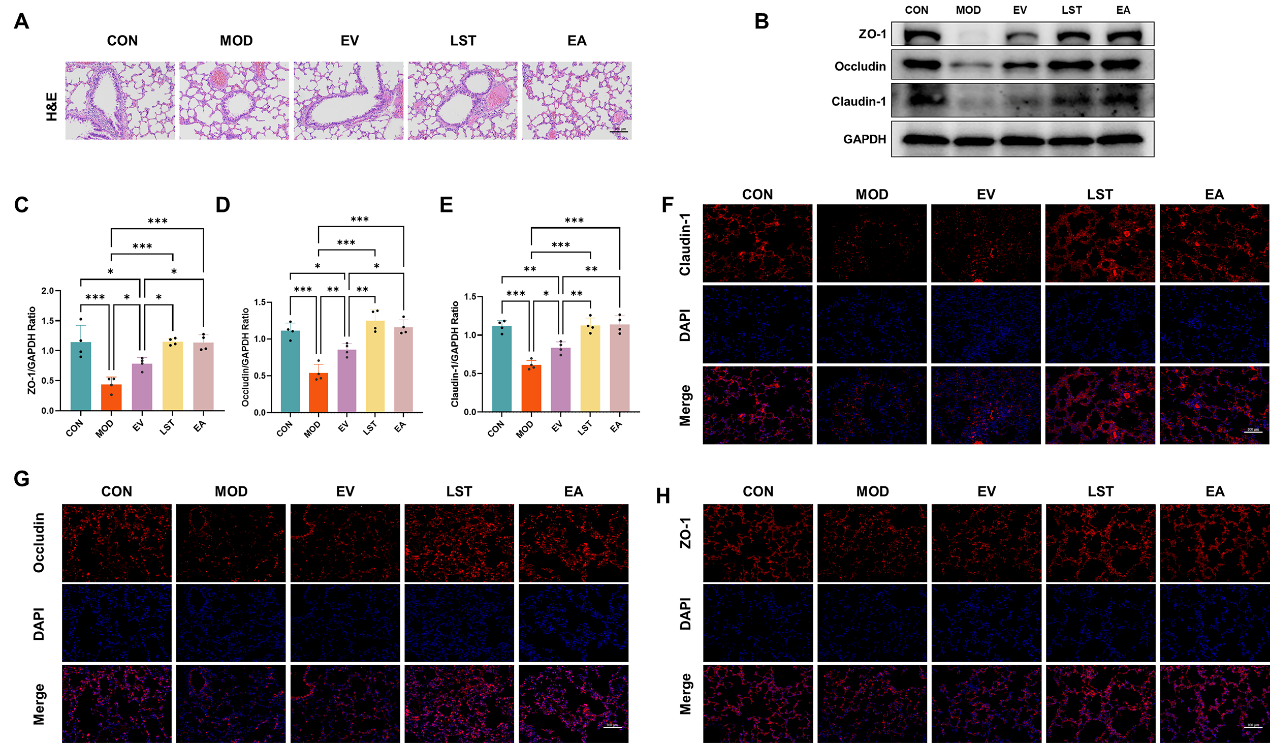


**Figure S8. Effects of EA on lung tissue integrity in VaD rats.** (A) H&E staining of lung tissue. Scale bar: 100 μm. (B-E) Protein expression of ZO-1, Occludin and Claudin-1 in lung tissue. (F-H) Immunofluorescence of ZO-1, Occludin and Claudin-1 in lung tissue. Scale bar: 100 μm. CON, normal control. MOD: VaD rats treated with normal saline every other day. EV: VaD rats administered EVs every other day. LST: VaD rats administered *L. salivarius* every other day. EA: VaD rats administered EAs every other day. Data are presented as mean ± SEM. **P* < 0.05, ***P* < 0.01, ****P* < 0.001.

**Reference:**

1. Yang Q, Chen Q, Zhang KB, Liu Y, Zheng JC, Hu DX, Luo J: **Sinomenine alleviates neuroinflammation in chronic cerebral hypoperfusion by promoting M2 microglial polarization and inhibiting neuronal pyroptosis via exosomal miRNA-223-3p.** *Acta Neuropathol Commun* 2025, **13:**48.

2. Zhang W, Xie J, Wang Z, Zhong Y, Liu L, Liu J, Zhang W, Pi Y, Tang F, Liu Z, et al: **Androgen deficiency-induced loss of Lactobacillus salivarius extracellular vesicles is associated with the pathogenesis of osteoporosis.** *Microbiol Res* 2025, **293:**128047.

3. Zheng C, Zhong Y, Zhang W, Wang Z, Xiao H, Zhang W, Xie J, Peng X, Luo J, Xu W: **Chlorogenic Acid Ameliorates Post-Infectious Irritable Bowel Syndrome by Regulating Extracellular Vesicles of Gut Microbes.** *Adv Sci (Weinh)* 2023, **10:**e2302798.

4. Yang J, He L, Dai S, Zheng H, Cui X, Ou J, Zhang X: **Therapeutic efficacy of sulforaphane in autism spectrum disorders and its association with gut microbiota: animal model and human longitudinal studies.** *Front Nutr* 2023, **10:**1294057.

5. Qiu J, Chi G, Wu Q, Ren Y, Chen C, Feng H: **Pretreatment with the compound asperuloside decreases acute lung injury via inhibiting MAPK and NF-kappaB signaling in a murine model.** *Int Immunopharmacol* 2016, **31:**109-115.

6. Zhang Y, Li H, Li B, Li Y, Chai X, Li S, Xue X, Li H, Zhao Y, Tang Y, et al: **Dachaihu decoction ameliorates abnormal behavior by regulating gut microbiota in rats with propionic acid-induced autism.** *Front Microbiol* 2025, **16:**1535451.

7. Zhang W, Zhong Y, Wang Z, Tang F, Zheng C: **Apple polysaccharide improves age-matched cognitive impairment and intestinal aging through microbiota-gut-brain axis.** *Sci Rep* 2024, **14:**16215.

8. Liu Q, Zhang L, Zhang J: **Induced pluripotent stem cell-derived neural progenitor cell transplantation promotes regeneration and functional recovery after post-traumatic stress disorder in rats.** *Biomed Pharmacother* 2021, **133:**110981.

9. Zhang S, Pan D, Chen S, Tang D, Yang H, Song D, Yuan B, Huang JH, Jiang M, Wang F, Xu Q: **Paeoniflorin ameliorates depressive behaviours by modulating microbiota-gut-brain axis functions.** *Gen Psychiatr* 2025, **38:**e101979.

10. Su SH, Wu YF, Lin Q, Zhang L, Wang DP, Hai J: **Fecal microbiota transplantation and replenishment of short-chain fatty acids protect against chronic cerebral hypoperfusion-induced colonic dysfunction by regulating gut microbiota, differentiation of Th17 cells, and mitochondrial energy metabolism.** *J Neuroinflammation* 2022, **19:**313.

11. Wang Z, Liu T, Liu L, Xie J, Tang F, Pi Y, Zhong Y, He Z, Zhang W, Zheng C: **Lactobacillus vaginalis alleviates DSS induced colitis by regulating the gut microbiota and increasing the production of 3-indoleacrylic acid.** *Pharmacol Res* 2025, **213:**107663.

12. Abbott NJ, Rönnbäck L, Hansson E: **Astrocyte-endothelial interactions at the blood-brain barrier.** *Nat Rev Neurosci* 2006, **7:**41-53.
